# De Novo Design of Inhibitors of DNA Methyltransferase 1: A Critical Comparison of Ligand- and Structure-Based Approaches

**DOI:** 10.3390/biom14070775

**Published:** 2024-06-28

**Authors:** Diana L. Prado-Romero, Fernanda I. Saldívar-González, Iván López-Mata, Pedro A. Laurel-García, Adrián Durán-Vargas, Enrique García-Hernández, Norberto Sánchez-Cruz, José L. Medina-Franco

**Affiliations:** 1 DIFACQUIM Research Group, Department of Pharmacy, School of Chemistry, Universidad Nacional Autónoma de México, Avenida Universidad 3000, Mexico City 04510, Mexico; pradodiana93@gmail.com (D.L.P.-R.); fer.saldivarg@gmail.com (F.I.S.-G.); pedro.a.laurel@gmail.com (P.A.L.-G.); 2 División Académica de Ciencias Básicas, Universidad Juárez Autónoma de Tabasco, Carretera Cunduacán-Jalpa de Méndez, Km 1, Cunduacán 86690, Tabasco, Mexico; papcorlm@gmail.com; 3 Instituto de Química, Unidad Mérida, Universidad Nacional Autónoma de México, Carretera Mérida-Tetiz Km. 4.5, Ucú 97357, Yucatán, Mexico; norberto.sanchez@iquimica.unam.mx; 4 Instituto de Química, Universidad Nacional Autónoma de México, Ciudad Universitaria, Mexico City 04510, Mexico; adv291882@gmail.com (A.D.-V.); egarciah@unam.mx (E.G.-H.); 5 Instituto de Investigaciones en Matemáticas Aplicadas y en Sistemas, Unidad Mérida, Universidad Nacional Autónoma de México, Sierra Papacál 97302, Yucatán, Mexico

**Keywords:** chemoinformatics, drug discovery, docking, epigenetics, epigenetic target profiler, focused libraries, fragments, library design, open-access

## Abstract

Designing and developing inhibitors against the epigenetic target DNA methyltransferase (DNMT) is an attractive strategy in epigenetic drug discovery. DNMT1 is one of the epigenetic enzymes with significant clinical relevance. Structure-based de novo design is a drug discovery strategy that was used in combination with similarity searching to identify a novel DNMT inhibitor with a novel chemical scaffold and warrants further exploration. This study aimed to continue exploring the potential of de novo design to build epigenetic-focused libraries targeted toward DNMT1. Herein, we report the results of an in-depth and critical comparison of ligand- and structure-based de novo design of screening libraries focused on DNMT1. The newly designed chemical libraries focused on DNMT1 are freely available on GitHub.

## 1. Introduction

The primary goal of de novo design is to generate new chemical entities with desired properties [1,2,3,4]. Generating bioactive compounds within the physicochemical-relevant chemical space is highly desirable in drug discovery. Thus, de novo design is an attractive approach to generating focused libraries with a desired or predicted bioactivity towards a biochemical or molecular target. Indeed, other than the common and traditional general screening compound libraries, chemical vendors and commercial companies are developing focused libraries of chemical compounds, as well as fragments (privileged fragments) focused on various targets of therapeutic relevance [5,6]. Although the physical samples (compound material) of the compounds are readily available for experimental screening in, for example, high-throughput or medium-throughput mode, the chemical structures are publicly accessible and can be used for benchmarking computational studies.

**Figure 1:**
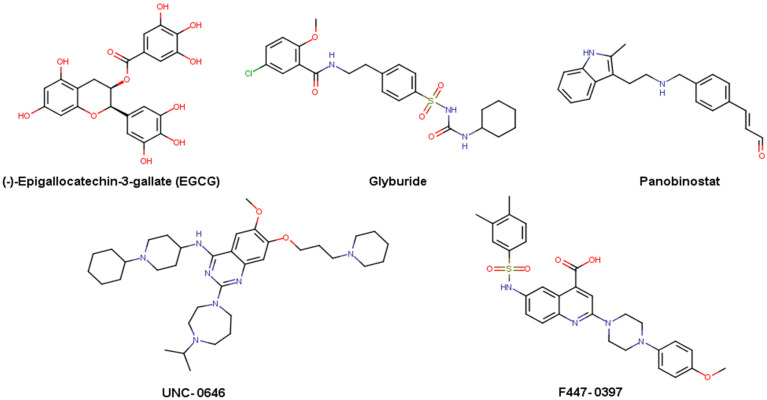
Chemical structures of representative inhibitors of DNA methyltransferases (DNMTs). Notably, F447-0397 was reported recently and has a novel chemical scaffold among DNMT1 inhibitors.

DNA methyltransferases (DNMTs) are one of the main epigenetic target families with clinical relevance [7,8,9]. DNMTs include two de novo methyltransferases, DNMT3A and DNMT3B, and the maintenance methyltransferase DNMT1 (the most abundant DNMT), which is in charge of duplicating the pattern of DNA methylation during replication, and it is necessary for adequate mammalian development. Two nucleoside inhibitors of DNMT methylation, azacitidine and decitabine, have been approved by the FDA to treat myelodysplastic syndrome [10]. Despite their high efficacy, nucleoside drugs suffer from undesirable pharmacokinetic profiles, chemical instability, and toxic side effects [11]. Due to DNA methylation being a fundamental mechanism for gene regulation, the design and development of non-nucleoside DNMT modulators is a promising strategy for developing novel epigenetic-based therapies. Among the earliest non-nucleoside inhibitors identified for DNMT1 is (-)-epigallocatechin-3-gallate (EGCG) (Figure 1). This compound and other DNMT1 inhibitors sourced from natural origins have been subject to extensive review [12,13].

In epigenetic drug discovery, including DNMT1, efforts are increasing to design and analyze focused libraries of DNMT inhibitors. Our research group recently reported a comprehensive analysis of the chemical content, diversity, and chemical space coverage of eleven commercial epigenetic-focused libraries with over 50,000 molecules. In that study, the most and least diverse chemical libraries were identified [14]. Moreover, separate research endeavors have identified five compounds, including glyburide and panobinostat (Figure 1), as DNMT1 inhibitors [15]. Also highlighted is the quinazoline UNC-0646 (Figure 1), initially identified as a G9a inhibitor, which has been recently discovered to also effectively inhibit DNMT1 at the nanomolar level [16].

Although in epigenetic drug discovery de novo design has been used to identify inhibitors of a bromodomain [17] and other epigenetic targets, such technique has been reported scarcely for designing DNMT inhibitors [18]. Recently, ligand-based de novo design based on compounds with reported activity for DNMT1 and a diverse subset of screening compounds were used to generate novel candidate DNMT1 inhibitors with drug-like properties. Then, the compounds designed de novo were used as reference for fingerprint-based similarity searching in a commercial epigenetic-focused library. The most similar compounds were acquired and tested in an enzymatic inhibition assay, identifying a DNMT1 inhibitor (F447-0397) with a novel chemical scaffold and an enzymatic inhibitory concentration in the micromolar range (Figure 1) [19]. The identification of F447-0397 as an inhibitor of DNMT1, inspired by de novo design, is the first approach to the potential of such drug designing approaches to identify novel active molecules with DNMT1 inhibitory activity. Also, recently Lanka et al. implemented a multi-step virtual screening involving docking a fragment library to yield two potential active compounds for DNMT1 [20].

To further explore the potential of de novo design to build epigenetic-focused libraries targeted toward DNMT1, we discuss a critical assessment of ligand- and structure-based de novo design approaches. The new chemical libraries focused on DNMT1 represent an addition to the epigenetic libraries currently available. The newly designed compound collections are freely available and can be used for further virtual and experimental screening.

## 2. Methods

**Figure 2:**
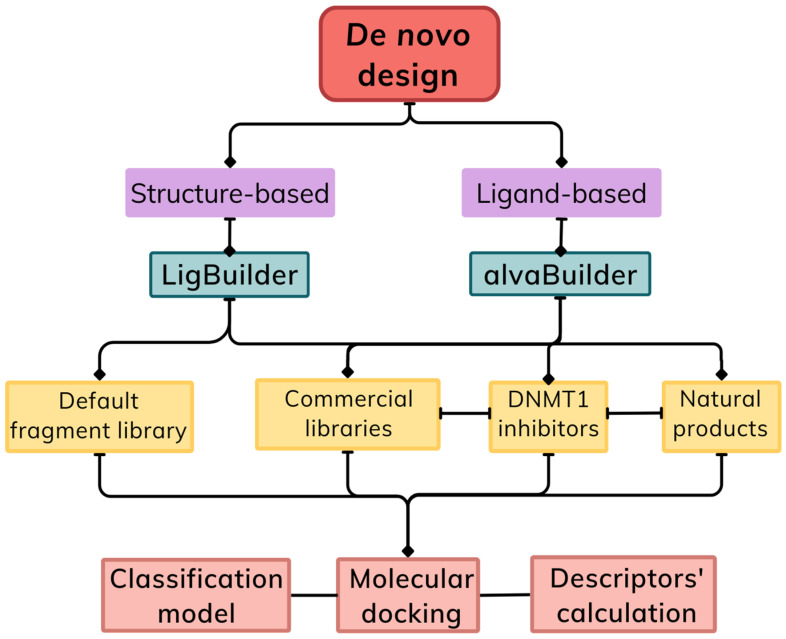
Overview of the de novo strategy comparison conducted in this study. Two main de novo design strategies were used, structure-based and ligand-based, and they were performed with the software LigBuilder and alvaBuilder, respectively. Both de novo compound libraries were profiled with machine learning classification models, molecular docking, and descriptors to characterize and compare the drug-likeness, synthetic accessibility, global structural, and property diversity, including the molecular scaffolds.

Figure 2 outlines the main de novo design approaches compared in this study. Briefly, the design was carried out using ligand-based (alvaBuilder [21]) and structure-based (LigBuilder [22]) strategies. The fragments’ sources were selected to represent similar starting points for both software, despite differences in the fragmentation procedure. alvaBuilder requires chemical libraries as training sets for its fragmentation. Then, generated fragments are used as building blocks by the software. Each molecule selected from the training set is split into fragments following the Bemis and Murcko rules [21]. In contrast, LigBuilder employs external fragment libraries provided by the user. In this study, fragment libraries were obtained using the fragmentation rules provided by the RECAP (retrosynthetic combinatorial analysis procedure) [23] algorithm or fragment libraries that are commercially available.

The fragments’ sources included DNMT1 inhibitors, commercial libraries, natural products, and default fragments in LigBuilder. Details of the filtration of chemical libraries and fragments are specified in the following sections. Designed molecules were compared to each other, taking into account the different fragment sources. To unify the comparison criteria, descriptors were calculated with the same methodology. The calculations performed were: quantitative estimate of drug-likeness (QED) [24], synthetic accessibility score (SAscore) [25], molecular docking with two docking programs, AutoDock Vina (Vina) [26,27], and LeDock [28]. We also profiled the newly designed libraries regarding scaffold content and the predictions with two DNMT1 classification models based on a recently developed model to estimate the activity of DNMT1 inhibitors [29] (Figure 2). In agreement with Open Science, the newly designed compound libraries and the code for all the analyses reported in this study are freely available on GitHub at https://github.com/DIFACQUIM/De-Novo_DNMT1 (accessed on 25 June 2024).

### 2.1. Data Curation

Chemical and fragment libraries and compounds designed de novo were curated using the same protocol, previously published [30,31], using RDKit open-source cheminformatics toolkit, version (2023.09.05) [32] and MolVS 0.1.1 [33] Python libraries. Briefly, compounds were standardized, and the largest fragment was kept in cases where molecules had more than one component. Only molecules with the elements: H, B, C, N, O, F, Si, P, S, Cl, Se, Br, and I were selected. The remaining compounds were neutralized and reionized to generate a canonical tautomer and remove duplicates. If present, the stereochemistry information was preserved only for the compounds designed de novo.

### 2.2. Ligand-Based De Novo Design

AlvaBuilder v.1.0.10 [21] was used for the ligand-based design. This software uses fragments from the user-selected training set to implement a graph-based construction of molecules. AlvaBuilder fragments the molecules from the training set into ring systems, linkers, and side chains. Eight different chemical libraries were used as training sets, as detailed in Section Training Chemical Libraries.

The scoring function was established with a range of seven descriptors (values summarized in Table S1 in Supplementary Materials) and a penalized substructure matching for nucleoside scaffolds (Figure S1). The score file with the set of rules is on GitHub at https://github.com/DIFACQUIM/De-Novo_DNMT1/tree/main/alvaBuilder (accessed on 25 June 2024). The ranges were established considering the mean and standard deviation values from molecules with reported biological activity against DNMT1, with IC_50_ ≤ 10 μM. This threshold has been previously applied in other studies to label active/inactive compounds [34,35].

Seven descriptors were computed with alvaDesc 2.0.10 [36]. They included molecular weight (MW), hydrogen bond donors (HBD) and acceptors (HBA), consensus partition coefficient octanol/water (logP), aqueous solubility (ESOL), synthetic accessibility (SAscore), and topological polar surface area (TPSA). The aggregation method was an arithmetic mean, and the same scoring function was used for all the training sets. The genetic algorithm was set with a population size of 70 and 100 iterations, generating 700 molecules for each training set.

#### Training Chemical Libraries

Eight compound libraries summarized in Table 1 were used as training sets for alvaBuilder, including five commercial libraries (two of which are epigenetic-focused), DNMT1 inhibitors, food chemicals from FooDB, and natural products. As the number of compounds available for each chemical library is highly variable, a filtration was made to match the number of 285 DNMT1 active compounds in ChEMBL. The first step after data curation was the selection of the molecules with MW greater than 300 to avoid fragment-like compounds [37]. The MW descriptor was computed and sorted in descending order with alvaMolecule 1.0.4 [38] after representing all the libraries with the Aromatic form available in the software. Then, a diverse subset of 285 molecules was selected with the MaxMin algorithm [39] implemented in Molecular Operating Environment (MOE) 2022.02, using the MACCS Keys fingerprint (166 bits) and the Tanimoto coefficient [40,41]. Details of the remaining number of compounds for each stage are in Table S2 in Supplementary Materials.

### 2.3. Structure-Based De Novo Design

LigBuilder V3 was used for the structure-based design [22]. LigBuilder constructs molecules by linking and growing fragments from the available libraries. Adding fragments to the default library or changing it completely is possible. The search strategy used in LigBuilder is a genetic algorithm to develop and evolve the molecules. This algorithm mimics the evolution of a population under selection pressure [22,51,52]. For the estimation of the binding affinity of the new molecules, the default empirical scoring function in LigBuilder was used in all cases.

#### 2.3.1. Binding Site Detection

To begin the calculations with LigBuilder, defining the binding site of the protein of interest is necessary. For this purpose, the software includes the CAVITY module, which analyzes the binding pocket of the protein’s three-dimensional (3D) structure of interest [53]. As a result of CAVITY, the required data of the binding pocket are ready for the construction step with the BUILD module (Section 2.3.2). The first step was to retrieve DNMT1 3D coordinates from the RCSB Protein Data Bank (PDB). The crystallographic structure of DNMT1 was PDB ID: 4WXX, available online: https://www.rcsb.org/ (accessed on 30 June 2023) [54]. Chain A from the crystallographic structure of DNMT1, without water molecules, was kept for the calculations, and all water molecules were eliminated. The calculations with CAVITY were in standard ligand mode, with the structure of *S*-adenosyl-*L*-homocysteine (SAH) used as a guide for the search of the binding cavities. The cavity with the best druggability prediction was chosen for the de novo design with all the fragment libraries.

#### 2.3.2. Molecule Design

Once the binding pocket was defined with CAVITY, the module BUILD was used in Exploring mode to design molecules with potential activity against DNMT1. Default parameters for the construction of molecules were modified for the descriptors: MW (175–665), logP (−3–7), hydrogen bond donors (0–8), and acceptors (1–12), to cover the previously observed values from DNMT1 inhibitors found in ChEMBL (minimum–maximum, Table S1). The genetic algorithm was set to a population of 500 and 10 generations. The configuration files modified for the construction of the molecules with LigBuilder are available on GitHub at https://github.com/DIFACQUIM/De-Novo_DNMT1/tree/main/LigBuilder (accessed on 25 June 2024).

#### 2.3.3. Fragment Libraries

Eight different fragment libraries were chosen as building blocks for LigBuilder, including the available default library, four commercial libraries (one epigenetic-focused), DNMT1 inhibitors, food chemicals from FooDB, and natural products. These were selected considering the sources of the training sets used before with alvaBuilder (Table 1). Notably, fragments from DNMT1 inhibitors, FooDB, and natural products were obtained from the fragmentation of the corresponding curated chemical libraries with the RECAP algorithm. Table 2 summarizes the sources and number of fragments of the eight libraries.

Since the number of fragments differs from the default library, the same number was selected from the remaining libraries. A workflow in KNIME 4.7.7 [55] was implemented to compute the different stages of the filtering. Fragments H_2_O, NH_3_, and HCl were not considered. The first stage was to choose all fragments with MW less than 300 Da, according to the Rule of Three (RO3) [37]. This was conducted with the RDKit Descriptor Calculation node.

**Figure 3:**
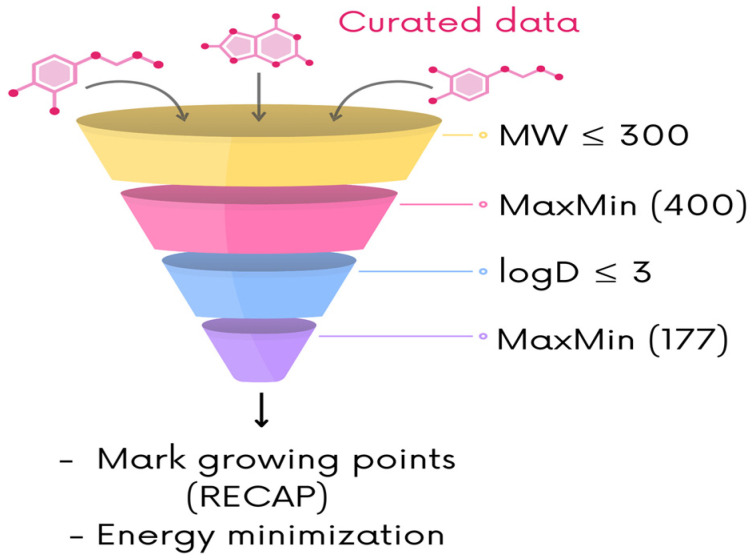
Filtration steps to select the fragments used as building blocks for LigBuilder. All the libraries matched the 177 fragments from the default library; their 3D coordinates were built, and the geometry was energy minimized for each one. The growing sites were specified only for the fragment libraries constructed with the RECAP algorithm.

Once the first filtering was made, a diverse subset of 400 fragments was selected with the MaxMin algorithm implemented in the RDKit Diversity Picker, using the MACCS Keys fingerprint (166 bits) and the Tanimoto coefficient [40,41]. Then, the distribution coefficient (logD), at pH = 7.4, of the 400 fragments was computed with ADMETlab 2.0 [56]. After that, the data were loaded into the KNIME workflow to keep the fragments with logD less or equal to three. Table S3 summarizes the details of the remaining number of fragments for each step. With the remaining compounds, a final diverse subset of 177 fragments was picked with the MaxMin algorithm, computed with MACCS Keys (166 bits) and the Tanimoto coefficient (Figure 3).

The default library from LigBuilder and the four commercial libraries (Table 2) were used with all the hydrogen atoms as growing points for the molecular assembly. The growing points for the three fragment libraries constructed with RECAP were selected, taking into account the site where the fragmentation was made. The RECAP fragment libraries were analyzed using DataWarrior 05.05.00 [57] to identify the fragmentation site. Using the molecule viewers BIOVIA Discovery Studio Visualizer 24.1.0.23298 [58] and PyMol 2.5 [59], the hydrogen atom or atoms where the fragmentation occurred were identified and marked as the growing site for each fragment.

**Table 2:** Fragment libraries used for the de novo design with LigBuilder.

Database	Description	Number of Fragments
LigBuilder default [22]	Common chemical groups and ring frameworks observed in organic compounds.	177
ChemDiv Fragments Library [60]	Fragments with desirable properties, including diversity.	11,269
ChemDiv Epigenetics Fragments [61]	Privileged fragments focused on epigenetic regulators.	9196
ChEMBL actives [23,45]	Fragments from ChEMBL actives (Table 1) obtained with RECAP.	1645
FooDB [23,46]	Fragments from FooDB compounds up to 1500 Da obtained with RECAP.	225,206
Life Chemicals Soluble Fragments [62]	Diversity-oriented fragment library with experimental solubility data.	1280
Selleckchem [63]	A collection of fragments for Fragment-Based Drug Discovery.	1015
UNPD-A [23,49,50]	Fragments from UNPD-A up to 1500 Da (Table 1) obtained with RECAP.	412,110

The 177 fragments of the final libraries were built, and their geometry was energy minimized using the MFF94x forcefield implemented on MOE 2022.02 [64] (Figure 3). Each minimized fragment was stored in an individual .mol2 file. The INDEX file of LigBuilder, to recognize the corresponding fragments for molecular construction, was modified for each of the libraries.

### 2.4. Visualization of the Chemical Space

To generate a visual representation of the chemical space of the de novo-designed libraries and reference datasets, we used t-distributed stochastic neighbor embedding (t-SNE) [65] and principal component analysis (PCA) based on structural fingerprints and continuous properties of pharmaceutical interest. The structural fingerprints used in this study were Extended Connectivity Fingerprints radius two (ECFP4) [66] and MACCS Keys (166 bits), which were computed for all molecules using the RDKit library [32]. Six molecular properties relevant to pharmaceuticals were computed: MW, HBA, HBD, TPSA, logP, and number of rotatable bonds (nRotB).

### 2.5. Drug Likeness and Synthetic Accessibility

We calculated the QED as an empirical and well-established measure of drug likeness. QED is based on eight molecular properties relevant to determining drug-likeness: logP, HBA, HBD, PSA, nRotB, number of aromatic rings, and number of structural alerts [24]. The weighted geometric mean of these properties is the resultant QED that goes from zero (worst) to one (best). The suggested threshold for the attractive compounds is 0.67. This value or higher is desirable for drug-like compounds. QED was calculated with RDKit, which is available in the Chem module.

The synthetic accessibility was computed using the SAscore. Synthetic accessibility is a combination of fragment structures comprising historical knowledge of chemical synthesis and a complexity penalty that considers non-standard structural features like stereocomplexity [25]. The score is scaled to 1 (very easy to synthesize) and 10 (very difficult to synthesize). Those molecules with a SAscore greater than six are considered not synthetically feasible. SAscore was calculated using the implementation available in the RDKit library.

### 2.6. Molecular Docking

Our research group previously published the molecular docking protocol used here, with RMSD re-docking values lower than 2 Å for the co-crystallized SAH [19]. This study used two docking programs with different algorithms, LeDock and Vina, to calculate docking scores for all the de novo-designed compounds. Relevant details of this protocol are explained in the following lines.

The same crystallographic structure of DNMT1 (PDB ID: 4WXX) used for binding site detection with CAVITY (Section 2.3.1) was selected for molecular docking calculations. The protein preparation was made with default settings of the QuickPrep module MOE v. 2022.02 [64]: addition of all the lacking hydrogen atoms, protonation state at pH 7, elimination of water molecules 4.5 Å farther from the protein, addition of missing amino acids residues (breaks of up to ten residues and terminal out gaps of up to five residues) and for larger gaps, neutralization of the endpoints adjoining empty residues and energy minimization. The parameters employed for the energy minimization stage were from the AMBER14:EHT forcefield (ff14SB [67] for the protein, MAB forcefield [68], and AM1-BCC charges for SAH [69]).

Before docking, the corresponding ligands (285 from the reference database and 8066 de novo compounds) were built, and their geometry was energy minimized using the MFF94x forcefield implemented on MOE. For every ligand, the most stable tautomer at physiological pH (7.4) was chosen [64].

#### 2.6.1. Docking with Vina

The file with the prepared ligands was split with the LeFrag module [28], and Open Babel v.3.1.1 [70] was used to convert to .pdb format. Protein and ligands were converted to .pdbqt with MGLTools v.1.5.6. The molecular docking was carried out with Vina v.1.2.3 [26,27] with an exhaustiveness of eight and five binding modes to output. The best score for each ligand was selected for further analysis with the code freely available at https://github.com/DIFACQUIM/Docking (accessed on 16 April 2024). The grid box was centered in the coordinates: −47.673, 61.885, 6.256 (x, y, z) with a search space of 17 × 25 × 14 Å.

#### 2.6.2. Docking with LeDock

Docking with LeDock [71] was carried out in the SAH binding site with the default settings: the grid centered 4 Å around the co-crystallized SAH. Twenty docking runs for every ligand and 1 Å for the root mean square deviation (RMSD) clustering. For further data analysis, the best score for every ligand was selected using the code available at https://github.com/DIFACQUIM/Docking (accessed on 16 April 2024).

### 2.7. Global Diversity and Scaffold Analysis

The total or global diversity of the de novo-designed libraries and the DNMT1 dataset was analyzed, considering multiple structure representations. Specifically, the datasets were compared regarding structural fingerprints, molecular scaffolds, and properties of pharmaceutical interest. Considering the different representations, the total or global diversity of the datasets was analyzed using consensus diversity plots (CDPlots) [72]. To assess a broader comparison, compounds from the DNMT1 dataset were retrieved from ChEMBL 33, all compounds were kept regardless type or magnitude of biological activity, and 743 compounds remained after curation.

#### Molecular Descriptors

The structural fingerprints used in this study to assess the global diversity were ECFP4 and MACCS Keys (166 bits), computed for all molecules using RDKit. Subsequently, a similarity matrix was generated based on these two fingerprints and the Tanimoto coefficient [73]. Values outside the diagonal of the similarity matrix were used to calculate the median MACCS Keys/Tanimoto of the pairwise comparisons.

The molecular scaffolds were generated using the Bemis–Murcko definition using RDKit [74]. We calculated the proportion of scaffolds relative to the number of compounds (N/M). Based on Cyclic System Retrieval (CSR) curves, we calculated the area under the curve (AUC) and the fraction of chemotypes that recover 50% of the molecules in the dataset (F_50_). The Shannon entropy of the ten most frequent scaffolds was also calculated [75]. To identify possible unique scaffolds, the unique scaffolds of each database were compared with each other.

Six molecular properties of pharmaceutical interest were computed to assess further diversity: MW, HBD, HBA, TPSA, nRotB, and logP. Subsequently, pairwise intra-database chemical diversity of the six molecular properties was determined using Euclidean distance.

### 2.8. Classification Models

Different classification models were developed to predict the activity class of compounds tested against DNMT1. Their construction process was based on the methodology previously described [29], which is summarized below.

#### 2.8.1. Dataset

Compounds with inhibitory activity against DNMT1 (reported as IC_50_) were obtained from ChEMBL 33 [76] (last updated May 2023). To build classification models, compounds were labeled as active if they had unequivocally assigned activities lower than or equal to 10 μM and as inactive in the opposite case. Compounds whose labels could not be unequivocally assigned were removed from the dataset (e.g., activity < 100 μM, activity > 1 μM, or duplicated compounds with contradictory labels). This resulted in a dataset containing 225 compounds, with 141 of them labeled as active and the remaining 84 labeled as inactive. This dataset was randomly divided in a stratified manner into two subsets: 80% was used to train different binary classification models and select the best ones using leave-one-out cross-validation (LOO-CV), and the 20% remaining was used as an external test set.

#### 2.8.2. Molecular Representations

Three molecular fingerprints of different designs were chosen as descriptors for the construction of the classification models: (a) Molecular ACCess System (MACCS) Keys (166-bit) [77], (b) Morgan fingerprint with radius 2 (2048-bit) [66], and (c) RDK fingerprint (2048-bit). All of them were generated using the RDKit open-source cheminformatics toolkit, version (2023.09.05) for Python.

#### 2.8.3. Machine Learning Methods

Binary classification models were built using five machine learning algorithms: k-Nearest Neighbors (k-NN) [78], Random Forest (RF) [79], Gradient Boosting Trees (GBT) [80], Support Vector Machines (SVM) [81], and Feed-Forward Neural Networks (FFNN) [82]. These methods were implemented using the Scikit-learn Python library (v1.4.1) [83]. Training instances were represented by feature vectors (fingerprints) and associated with class labels (active/inactive). Hyperparameters for each model were optimized using leave-one-out (LOO) cross-validation over a limited space. Only select hyperparameters for each algorithm were optimized to keep the search space small. The considered hyperparameters for each model are included in Table S4 in Supplementary Materials, with default values assumed if not explicitly indicated.

Each optimized model was defined as the combination of an algorithm and a fingerprint, whose hyperparameters were optimized using LOO-CV, with balanced accuracy (BA) employed to select the best hyperparameters. To estimate the confidence of the predictions as they differ from the training set, the best-performing models were also compared in terms of their precision and recall computed on a distance-to-model (DM) basis [84,85] using Morgan fingerprints. For that, predictions were categorized into four quartiles according to their mean Jaccard distance to the compounds in the training set. Having the confidence estimation, these models were retrained in the entire dataset and applied to predict the activity class of the compounds generated by de novo design.

### 2.9. Molecular Dynamics

MD simulations were conducted using the crystal structure of DNMT1 (PDB ID: 4WXX, resolution 2.6 Å [86]). The simulations were performed with the pmemd.cuda module of AMBER 22, employing the FF19SB and ZAFF forcefields, and the OPC water model [87,88,89]. Detailed simulation protocols followed methods described previously [90,91]. The crystal structure of the enzyme was prepared for simulations by adding missing loops using MODELLER software version 10.4 [92]. Simulations for three DNMT1 complexes with designed molecules were conducted at 310 K, pH 7.3. Ligand topology and parameter files were generated with the Antechamber suite and the general Amber force field (GAFF2) for organic ligands [93,94]. Atomic charges were derived using the AM1-BB method [69]. Five 100 ns replicas for each complex were produced. Structural analysis was performed using CPPTRAJ [95].

## 3. Results and Discussion

In the following sections, results from the construction and cheminformatic analysis of two de novo design-focused libraries on DNMT1 are described (strategy outlined in Figure 2). Docking scores and results from two classification models are also discussed as an insight into the potential activity of the designed libraries. Since information on the 3D coordinates of DNMT1 and compounds with reported activity against this target are available, we used ligand- and structure-based strategies. The comparison of both strategies could disclose the potential differences between the molecular and structural characteristics of the libraries.

The ligand-based strategy was conducted with alvaBuilder, restricted with the scoring function to propose molecules with properties within the observed value ranges for the selected descriptors. The ranges were established by considering the values of known DNMT1 inhibitors. The curated library contains 5575 compounds from eight different training sets used as fragment sources. The structure-based strategy was performed with LigBuilder, restricted to a binding site cavity surrounding SAH. The curated library has 2491 compounds from eight distinct fragment libraries.

### 3.1. Ligand-Based De Novo Design with alvaBuilder

**Figure 4:**
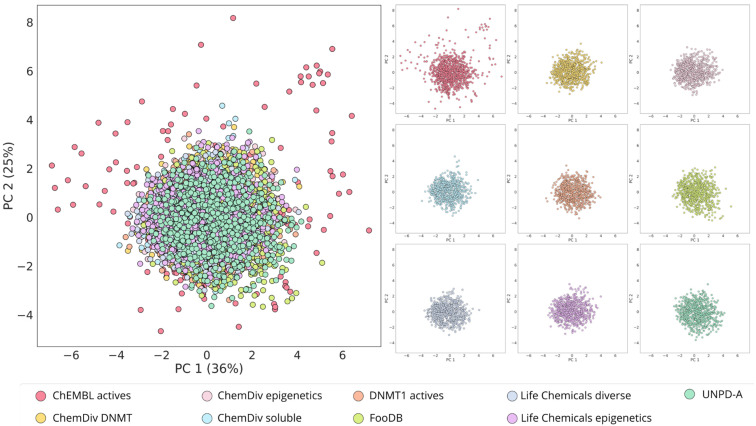
Chemical space visualization of the de novo-designed libraries with alvaBuilder. The visual representation was made with PCA as dimensionality reduction and six physicochemical properties as molecular representation. On the left are the nine superimposed databases, and on the right are the individual datasets using the same coordinates as the corresponding representations on the left.

**Figure 5:**
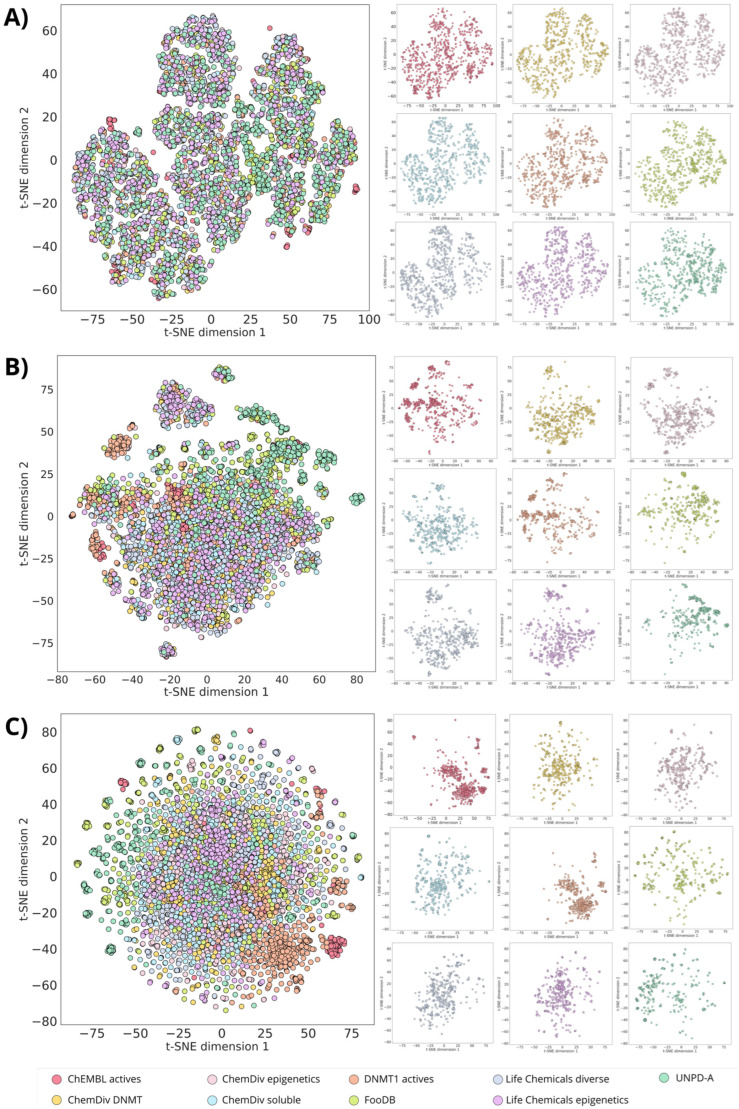
Chemical multiverse visualization of the de novo-designed libraries with alvaBuilder. The visual representations were generated using t-SNE and **A**) drug-like properties, **B**) MACCS Keys (166 bits), and **C**) ECFP4 (1024 bits) as molecular representations. On the left are superimposed databases, and on the right are the individual datasets using the same coordinates as the corresponding representations on the left.

One of the desired characteristics of the focused libraries on DNMT1, here constructed with de novo design, is to preserve the chemical similarity in terms of physicochemical properties while exploring different regions of the chemical space based on molecular structure. From the chemical space visualizations based on six molecular properties of pharmaceutical relevance (Figure 4 and Figure 5A), it can be seen that compounds designed with alvaBuilder are within the property space of the molecules with reported activity against DNMT1 on ChEMBL. This conclusion can be derived from both visualizations generated with PCA and t-SNE. To sum up, the observation could be related to the scoring function used in alvaBuilder (Section 2.2), based on almost the same molecular properties selected for the chemical space visualizations. Five of six properties were also included in the scoring function, only excluding nRotB. The scoring included ESOL and SAscore results and a penalty for substructures commonly found in analog nucleosides. The latter consideration was utterly relevant since the goal is expanding the chemical space of DNMT1 non-nucleoside inhibitors. The properties of the scoring function for alvaBuilder are constraints for the design and could be causing the visualization of all the design libraries to be more compact in the PCA graphs (Figure 4).

In contrast, the visualizations based on molecular fingerprints, MACCS Keys, and ECFP4 (Figure 5B,C) suggest that de novo compounds designed with alvaBuilder populate different areas of the chemical space, which is more noticeable when compounds are represented with the ECFP4 fingerprint. Interestingly, compounds that were designed with information about compounds published in ChEMBL (DNMT1 actives, depicted in orange) are in the same coordinates of the reference database for both fingerprints.

The compounds designed from the three ChemDiv libraries and the two Life Chemicals libraries (Table 1) cover similar regions of the chemical space with the t-SNE based on MACCS Keys (Figure 5B). These observations are expected because chemical libraries from the same provider could share structural features detected with the MACCS Keys. However, differences are observed with the visualization based on ECFP4 (Figure 5C), associated with the differences in resolution between the two fingerprints. Compounds from FooDB and UNPD-A cover similar areas with both representations; the reason for this could be the account of the rest of the libraries being focused on small, less complex molecules.

**Figure 6:**
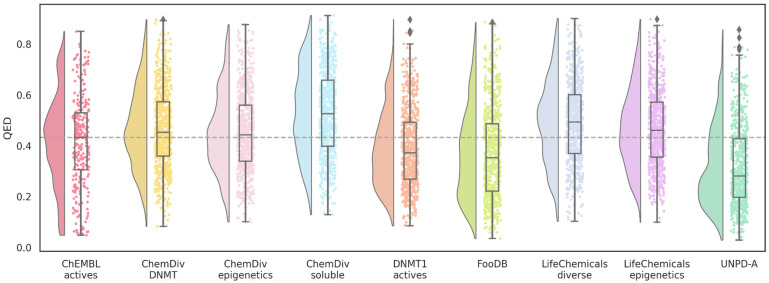
Distribution of QED values of de novo compounds designed with alvaBuilder. Each source of fragments is represented in a different color. Active compounds from ChEMBL are used as a reference (pastel red), and the mean value of these compounds is marked with a dotted gray line.

Distribution of the QED values (Figure 6) showed that the median values of all databases, even the ChEMBL’s DNMT1 inhibitors used as a reference, do not comply with the suggested threshold of 0.67 or higher. In this case, the mean from ChEMBL actives will be more relevant because the aim is to find new hits for DNMT1. Since hits are considered for earlier stages of drug discovery, biological activity normally carries more relevance than drug-like properties or potency. This could explain the lower values from known inhibitors with reported activity in ChEMBL. Nevertheless, QED values are useful for ranking and prioritizing the synthesis of de novo compounds or libraries.

Remarkably, most of the designed databases have similar or better QED mean values than the ChEMBL’s DNMT1 inhibitors. Molecules designed with DNMT1 inhibitors as the source of fragments decreased slightly. However, FooDB and UNPD-A molecules have a more pronounced change in their mean values. These could be inherent to the tendency of larger molecular complexity commonly encountered in natural products and food chemicals as compared to small drug-like molecules, represented by all other training sets. Thus, indirect evidence of alvaBuilder compounds inheriting structural characteristics from the building blocks is shown with these results.

**Figure 7:**
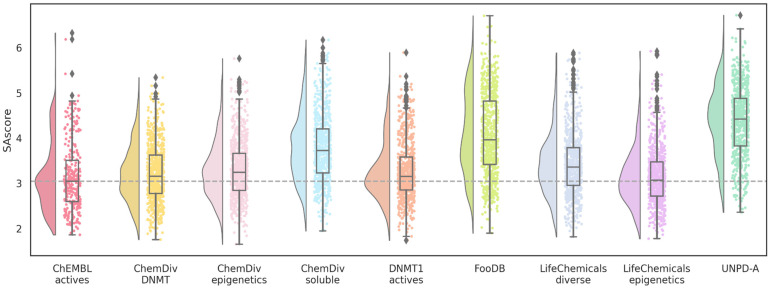
Distribution of SAscore values of de novo compounds designed with alvaBuilder. Each source of fragments is represented in a different color. Active compounds from ChEMBL are used as a reference (pastel red), and the mean value of these compounds is represented by a dotted gray line.

Similarly, SAscore values for five datasets are close to the reference; molecules designed from ChemDiv soluble, FooDB, and UNPD-A present higher means (Figure 7). This result could be associated with the complexity penalty of SAscore. In the case of ChemDiv soluble, another hypothesis is that the fragments could be more difficult to obtain because solubility issues are usually part of the optimization process. Of note, only 19 molecules of the 5575 (0.34%) have a SAscore greater than six.

**Figure 8:**
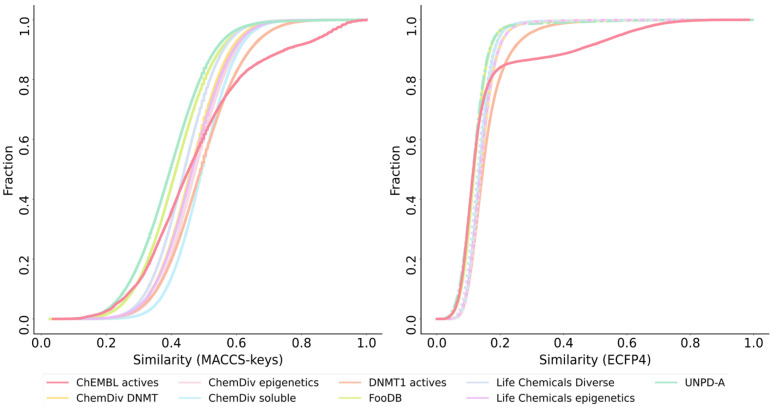
Cumulative distribution functions of the pairwise similarity values are computed with the Tanimoto coefficient and MACCS Keys 166 bits (**left**) and ECFP4 fingerprints (**right**) for the de novo libraries designed with alvaBuilder.

To compare the structural diversity of the generated databases from different fragment sources, the Tanimoto coefficient was used along with MACCS Keys (166 bits) and ECFP4 (1024 bits) fingerprints. According to MACCS Keys, molecules from UNPD-A are the most diverse, followed by FooDB and Life Chemicals diverse, which can be seen in the cumulative distribution functions (Figure 8) and values for mean and median similarity (Table S5 in Supplementary Materials).

UNPD-A is also the most diverse according to the ECFP4 fingerprint, followed closely by the ChEMBL’s DNMT1 inhibitors and molecules designed from FooDB (Table S6). ECFP4 similarity is considerably lower and has very close values among the nine databases, evidenced by the proximity of the curves (Figure 8). This is expected because ECFP4 codifies connectivity features along the selected radius, while MACCS Keys has a pre-defined list of features, giving the first more resolution [96].

**Figure 9:**
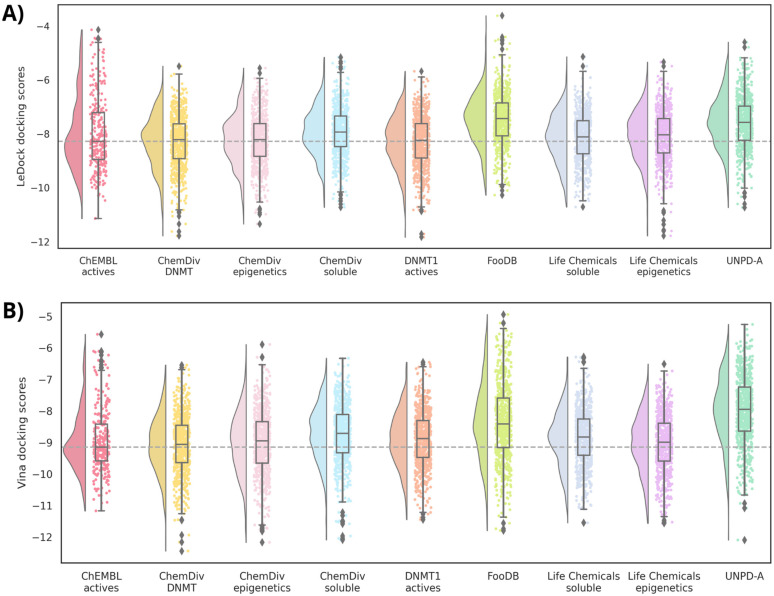
Raincloud plots summarizing the docking scores profile computed with **A**) LeDock and **B**) Vina for all compound datasets designed with alvaBuilder. Each source of fragments is represented in a different color. Active compounds from ChEMBL are used as a reference (pastel red). The mean value of these compounds is also marked with a dotted gray line.

In the case of molecular docking scores, the nine alvaBuilder databases have median values around the reference database for LeDock, as well as for Vina (Figure 9). Compounds designed from FooDB and UNPD-A present less auspicious medians. We have observed that ligand efficiency (LE), computed as the docking scores divided by the number of heavy atoms, improved the correlation between docking scores and biological activity. For that, LE values were calculated, and the results can be found in Tables S9 and S10. While means of FooDB and UNPD-A tend to the value of ChEMBL inhibitors with Vina LE, LeDock LE keeps the same trend as the scores.

### 3.2. Structure-Based De Novo Design with LigBuilder

**Figure 10:**
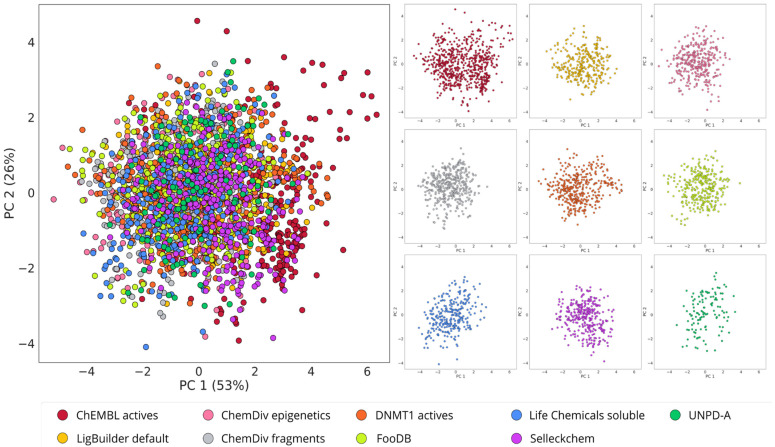
Chemical space visualization of the de novo-designed libraries with LigBuilder. The visual representation was made with PCA as dimensionality reduction and six physicochemical properties as molecular representation. On the left are the nine superimposed databases, and on the right are the individual datasets using the same coordinates as the corresponding representations on the left.

**Figure 11:**
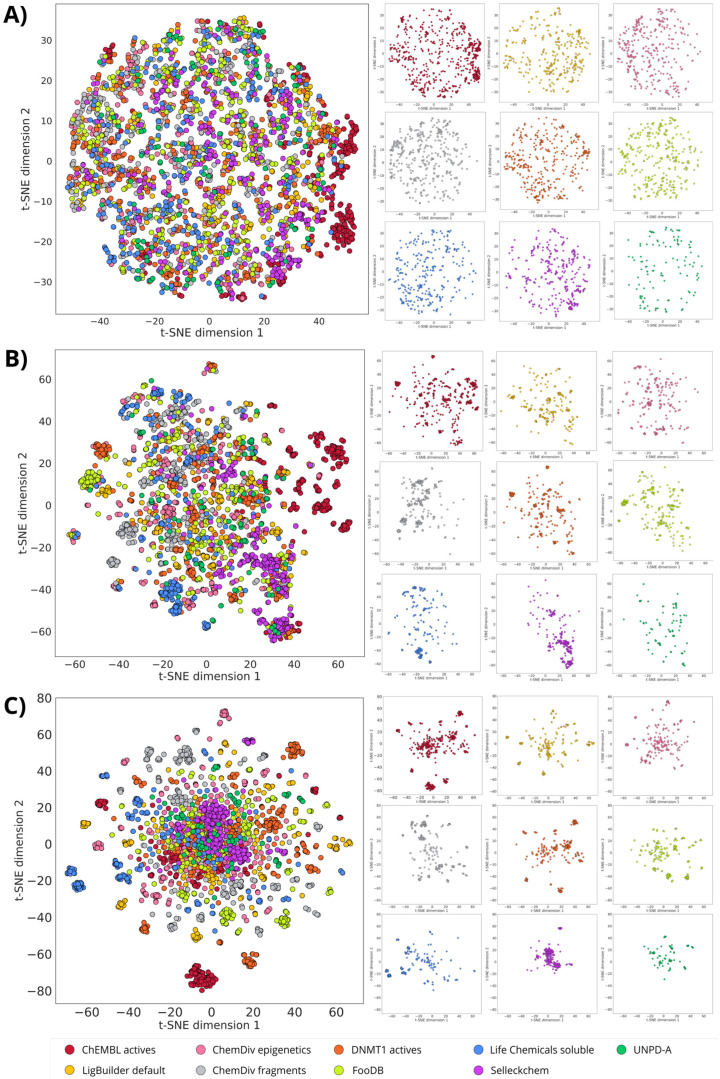
Chemical multiverse visualization of the de novo-designed libraries with LigBuilder. The visual representations were generated using t-SNE and **A**) drug-like properties, **B**) MACCS Keys (166 bits), and **C**) ECFP4 (1024 bits) as molecular representations. On the left, the nine databases are superimposed, and on the right are the individual datasets using the same coordinates as their corresponding representations on the left.

As detailed in the Methods, the molecular construction in LigBuilder was restricted by the characteristics of the binding pocket found with CAVITY. MW, logP, HBA, and HBD values from ChEMBL’s DNMT1 inhibitors were also considered in the design. Both constraints were necessary to control the molecular size of the compounds designed. As a result, the chemical space visualizations based on drug-like properties (Figure 10 and Figure 11A) show that the nine databases share the property-based chemical space, similar to the alvaBuilder compounds. Compounds from LigBuilder appear more diverse than the compounds designed with alvaBuilder. These results could be due to the limits of the molecular descriptors because the scoring function of alvaBuilder was more restricted with the median and standard deviation, while ranges for LigBuilder included minimum and maximum descriptor values.

Likewise, designed compounds from ChEMBL’s DNMT1 inhibitors share the same areas of the reference database with the representation of both fingerprints, although exhibiting some distinct clusters (Figure 11). In general, molecules from the five commercial libraries exhibit distinguishable areas between all of them, both with MACCS Keys (Figure 11B) and ECFP4 (Figure 11C). Also, for this library, FooDB compounds are populating similar areas to those designed from DNMT1 inhibitors, unlike alvaBuilder molecules that share more space with UNPD-A compounds. In contrast, molecules designed from UNPD-A fragments are less spread than the alvaBuilder ones. This could be due to a reduction in the number of molecules for this dataset. Notably, Selleckchem compounds have a tighter cluster than the rest of the databases, especially for the visualization with ECFP4 (Figure 11C).

**Figure 12:**
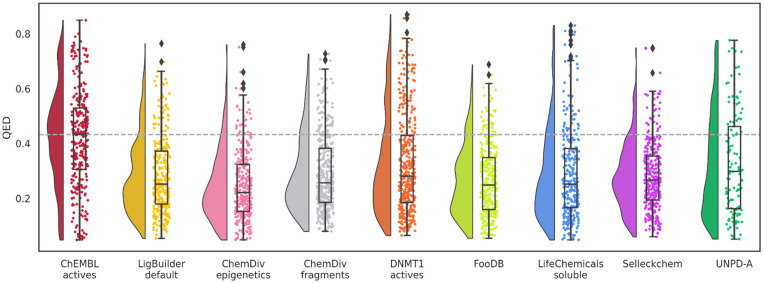
Distribution of QED values of de novo compounds designed with LigBuilder. Each source of fragments is represented in a different color. Active compounds from ChEMBL are used as a reference (wine red); the mean value of these compounds is also marked with a dotted gray line.

The distribution of the QED values indicates that the eight databases have lower QED medians than the reference database and, consequently, less drug-like properties (Figure 12). Furthermore, the point distribution (one point represents one molecule) and the half-violin from the raincloud plot tend to have even lower QED values than the median. Since the visualization of chemical space based on drug-like properties indicated that these datasets populated similar areas, the decrease could be associated with the number of aromatic rings or structural alerts of QED. The increase in molecular complexity could be due to the characteristics of the binding cavity of DNMT1 since LigBuilder constructs the compounds in the binding site.

**Figure 13:**
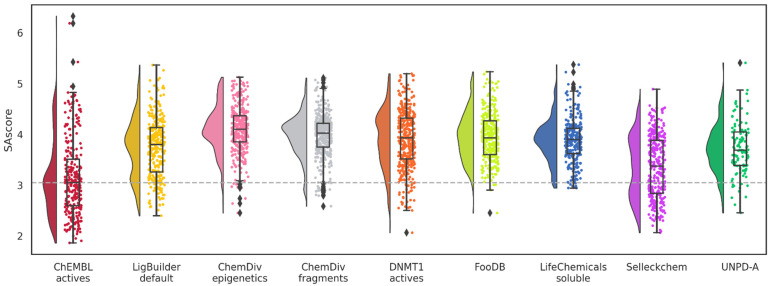
Distribution of SAscore values of de novo compounds designed with LigBuilder. Each source of fragments is represented in a different color. Active compounds from ChEMBL are used as a reference (wine red). The mean value of these compounds is represented with a dotted gray line.

Equally, the nine databases have less favorable scores regarding the distribution of SAscore values (Figure 13). However, unlike the previous QED scores, most point distributions tend to cluster around the mean values. An increase in SAscore is expected and has been addressed as one of the issues with de novo design. Although the synthetic feasibility could be challenging, all the molecules are below the suggested threshold of six.

**Figure 14:**
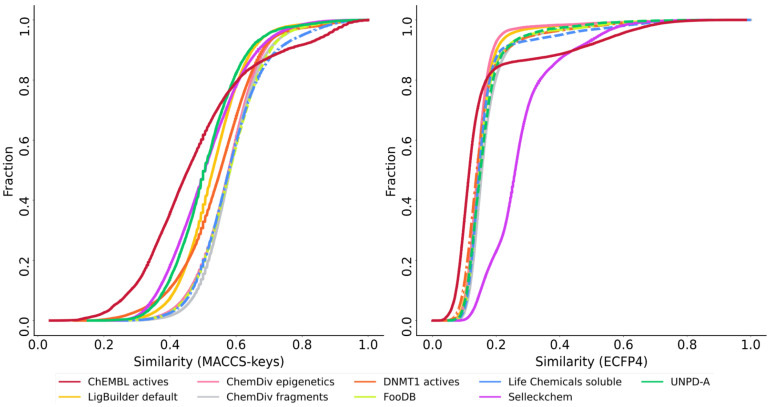
Cumulative distribution functions of the pairwise similarity values computed with the Tanimoto coefficient and MACCS keys 166 bits (**left**) and ECFP4 fingerprints (**right**) for the de novo libraries designed with LigBuilder.

For the cumulative distribution functions computed with the Tanimoto coefficient and two fingerprints (MACCS Keys and ECFP4) (Figure 14), we found that the reference database of ChEMBL actives was the most diverse in both instances as confirmed by the median similarity values (Tables S7 and S8 in Supplementary Materials). In the case of MACCS Keys, the following most diverse datasets were molecules from UNPD-A and Selleckchem. In contrast, molecules from the Selleckchem library were the least diverse, with ECFP4 as molecular representation. The most diverse database with this last fingerprint, after ChEMBL actives, were compounds constructed from this reference database (DNMT1 actives) and ChemDiv epigenetics.

**Figure 15:**
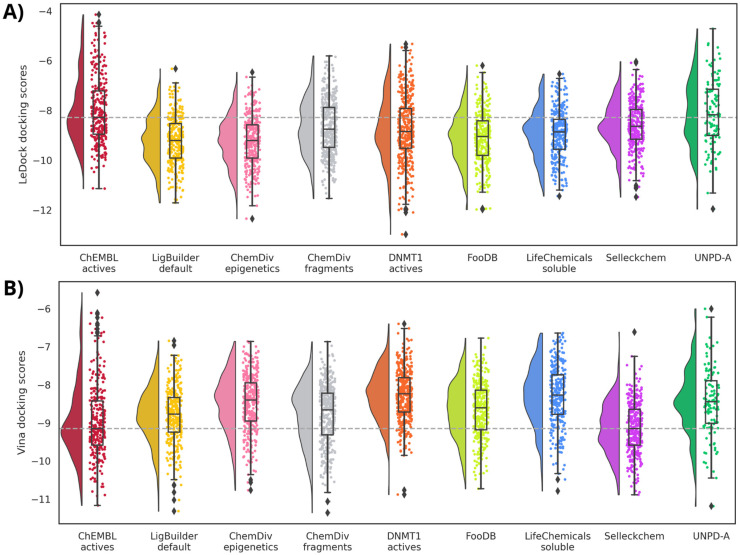
Raincloud plots summarizing the docking scores profile computed with **A**) LeDock and **B**) Vina for all compound datasets designed with LigBuilder. Each source of fragments is represented in a different color. Active compounds from ChEMBL are used as a reference (wine red). The mean value of these compounds is also marked with a dotted gray line.

Regarding the molecular docking scores, mean values are equal to or better than the computed scores for ChEMBL’s DNMT1 inhibitors for the case of LeDock (Figure 15A). In contrast, the eight designed databases have less favorable scores with Vina, except for Selleckchem compounds that equal the reference median. Although the numerical variations are around one point, these differences are not considered significant. LE values (Tables S11 and S12 in Supplementary Materials) also do not have marked changes in the numerical values.

### 3.3. Global Diversity and Scaffold Analysis

**Figure 16:**
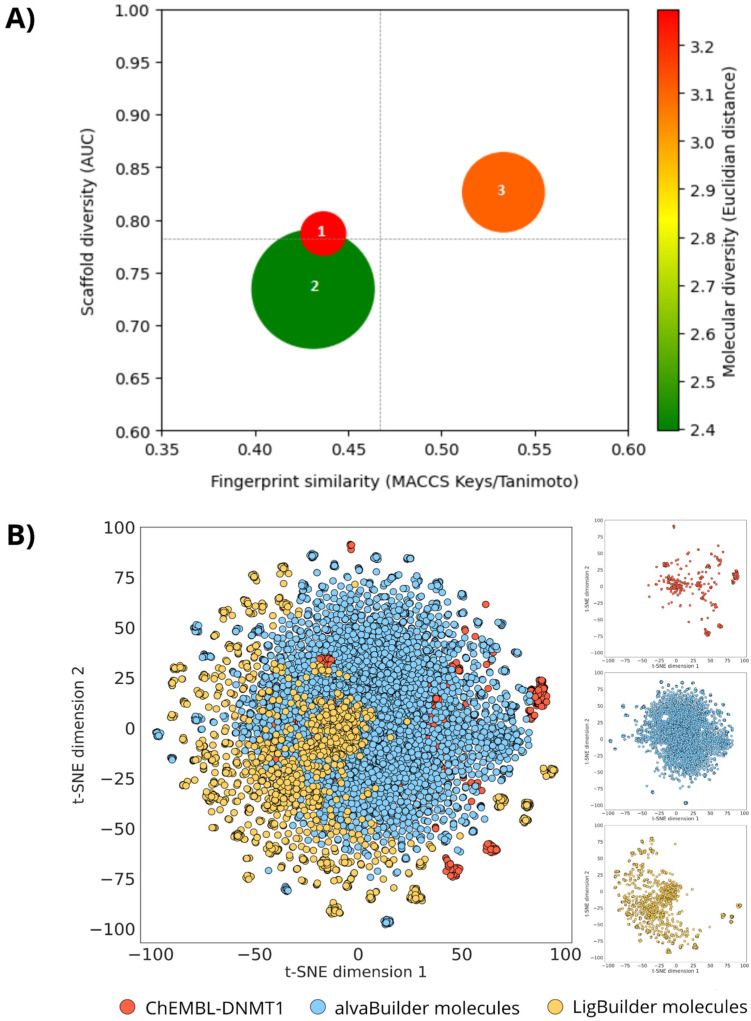
**A**) Consensus Diversity Plot comparing the diversity of DNMT1 dataset (1) and de novo compounds generated by alvaBuilder (2) and LigBuilder (3) software. The median similarity computed with MACCS Keys and the Tanimoto coefficient of the dataset is plotted on the X axis and the AUC of the scaffold recovery curves on the Y axis. Data points are colored by the diversity of the physicochemical properties of the dataset, as measured by the Euclidean distance of six properties of pharmaceutical relevance. The distance is represented with a continuous color scale from red (more diverse) to orange/brown (intermediate diversity) to green (less diverse). The data point’s size represents the dataset’s relative size: smaller data points indicate compound datasets with fewer molecules. **B**) Chemical space visualization with t-SNE as dimensionality reduction technique and ECFP4 as molecular representation. Each dataset is depicted in different color.

As described in Section 2, we compared the diversity of the de novo-designed libraries and the DNMT1 dataset in terms of fingerprints, molecular scaffolds, and properties of pharmaceutical interest employing CDPs [72]. Figure 16 shows a comparison of the overall (global) structural diversity of all three datasets, Figure 16A plots a CDP considering four parameters described in Table 3, and Figure 16B shows a representation of the chemical space based on ECFP4. Every point in the CDP corresponds to a single dataset. The median corresponding to each dataset, computed with MACCS Keys/Tanimoto, is plotted on the X-axis. The area under the curve (AUC) for the scaffold recovery curves is plotted on the Y-axis. The size of the data points represents the relative sizes of each dataset, and the color of each data point represents the diversity of molecular properties.

According to Figure 16A, the compounds designed by alvaBuilder were the most diverse as measured by fingerprints, which assess diversity based on complete structures. Regarding scaffold diversity, compounds designed by alvaBuilder were also the most diverse, followed by the DNMT1 dataset and compounds generated by LigBuilder. Furthermore, compounds designed with LigBuilder were the most diverse in terms of molecular properties, only behind the DNMT1 compounds. As expected, compounds designed using a ligand-based approach (alvaBuilder) had the lowest property-based diversity because of the used scoring function that considers drug-like properties to generate compounds.

In the evaluation of the structural diversity among the three databases based on the representation of the ECFP4 chemical space (Figure 16B), it is observed that the compounds generated by alvaBuilder share regions of the chemical space similar to the DNMT1 dataset. In contrast, compounds generated by the LigBuilder program occupy less densely populated regions of chemical space compared to other databases. It is important to note that no duplicate structures were observed within this intersection.

**Figure 17:**
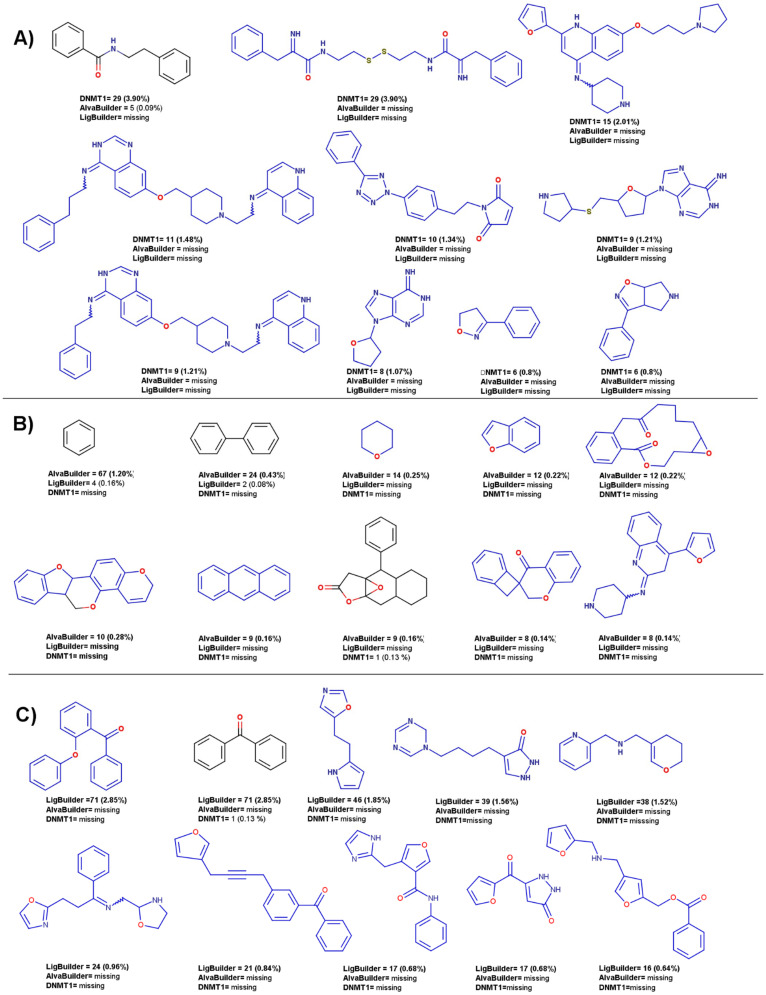
Ten most frequent scaffolds from **A**) DNMT1 dataset and libraries designed with **B**) alvaBuilder and **C**) LigBuilder. The presence and frequency in each of the three datasets is indicated. Unique scaffolds for each set of compounds are indicated in blue.

Table 3 further summarizes additional metrics of scaffold diversity, such as the ratio (N/M), F_50,_ and Scaled Shannon Entropy (SSE). Unlike the CSR curves, which assess the diversity of entire datasets, SSE measures the scaffold diversity of the most populated scaffolds. The SSE value ranges from 0, indicating uniformity in chemotype distribution across all compounds (minimum diversity), to 1.0 when all the compounds are evenly distributed among the n acyclic and/or cyclic systems (maximum diversity). According to SSE values depicted in Table 3 for the ten most frequent scaffolds, compounds designed with LigBuilder are the most diverse, followed by DNMT1 molecules and compounds designed by alvaBuilder. Figure 17 depicts an overview of the ten most frequent scaffolds from each database. Unique scaffolds for each database are highlighted in blue. The prevalence of acyclic compounds was notably higher in DNMT1 dataset (5.24%) and compounds designed by alvaBuilder (2.76%). LigBuilder, conversely, generated only 19 (0.76%) acyclic compounds. Among the 472 scaffolds identified in the DNMT1 molecules, 22 are present in compounds generated by alvaBuilder, five in compounds generated by LigBuilder, and three are shared between both libraries.

### 3.4. Classification Models

Fifteen optimized binary classification models were constructed to predict the activity class of compounds tested against DNMT1. These models resulted from training five machine learning algorithms using three different molecular fingerprints as descriptors as detailed in Section 2. Each model is represented as a combination of a fingerprint and algorithm (e.g., algorithm + fingerprint). Hyperparameters for each model were optimized through an exhaustive search employing LOO-CV with BA as the performance metric for selecting the best set of hyperparameters.

Overall, most of the optimized models exhibited strong performance, with a mean BA score exceeding 0.6, with the models SVM + RDK and FFNN + Morgan having the best performance on the training set with BA values of 0.849 and 0.847, respectively, and consistent performances on the external test set with BA values of 0.775 and 0.793, respectively. Table S13 in Supplementary Materials includes summary statistics for all models.

Although BA is suitable for assessing model performance on imbalanced datasets, correctly identifying active compounds is prioritized in practical medicinal chemistry applications. Therefore, individual models were evaluated in terms of their precision and recall, which represents the model’s ability to correctly classify active compounds, computed on a DM basis as detailed in Section 2. These results are summarized in Table 4.

For both models, precision and recall values decrease as long as the predicted compounds become more distant from those in the training set. However, even for distant compounds (Q3 and Q4), precision remains above 0.6, which is better than a random guess considering the data imbalance on each quartile (20/45 for Q3 and 9/45 for Q4). In terms of recall, the FFNN + Morgan model shows the best performance in Q3 and Q4, making it the best choice for selecting active compounds distant from those in the training set. Distance-to-model performance for the test set is included in Table S14, which shows a similar behavior. However, considering the limited number of compounds on each quartile, they tended to be over-optimistic (e.g., perfect precision at Q4) and were not considered for the final classification of compounds.

Both models were retrained in the entire dataset and applied to predict the activity class of the compounds generated by de novo design, and their DM was calculated. The number of compounds predicted as active for each quartile and dataset are summarized in Table 5 and Table 6 for SVM + RDK and FFNN + Morgan, respectively. Overall, most of the compounds generated by de novo design were distant to the training set, with just two of them falling in Q2, 63 of them falling in Q3, and 7842 falling in Q4.

**Figure 18:**
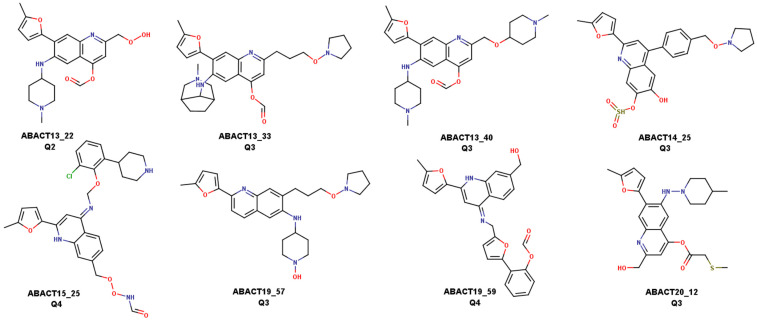
Chemical structures of the eight compounds predicted as active by the two classification models.

The two models predicted eight compounds as active; their chemical structures are shown in Figure 18. Significantly, all have “long or extended scaffolds”, a feature highlighted for active molecules against DNMT1 [15,97,98]. Furthermore, the eight molecules are quinolines with similar substitution patterns, and this family of compounds has been reported before by several research groups focused on DNMT inhibitors [99,100,101,102].

Moreover, 159 compounds obtained mean Jaccard distances higher than those observed for Q4, which involves an unknown confidence in their predictions. The SVM + RDK model predicted only 15 compounds as active against DNMT1, all generated by alvaBuilder, particularly from the ChemDiv DNMT dataset. On the other hand, the FFNN + Morgan model predicted active compounds for all groups of compounds generated by de novo design, with marked differences.

For all the groups generated by alvaBuilder, the proportion of compounds predicted as active was higher than 0.13, with DNMT1 actives and UNPD-A being the datasets with higher proportions, while for compounds generated by LigBuilder, this proportion was always below 0.10. This observation could be associated with the differences in fragment acquisition for each software. Namely, alvaBuilder incorporates an internal fragmentation algorithm based on Bemis–Murcko scaffolds, while LigBuilder takes fragments directly from the library.

As a result of the alvaBuilder fragmentation, the information about the position of the linkers is kept, so the complete molecules can be reconstructed from their corresponding fragments. This preserves chemical knowledge inherent to the scaffold and the substitution pattern in the fragments. In contrast, information about the original substitution of a fragment in LigBuilder has to be user-defined as a growing site. That information was not disclosed for the selected fragment libraries and was only available for the RECAP fragments.

### 3.5. Molecular Dynamics

**Figure 19:**
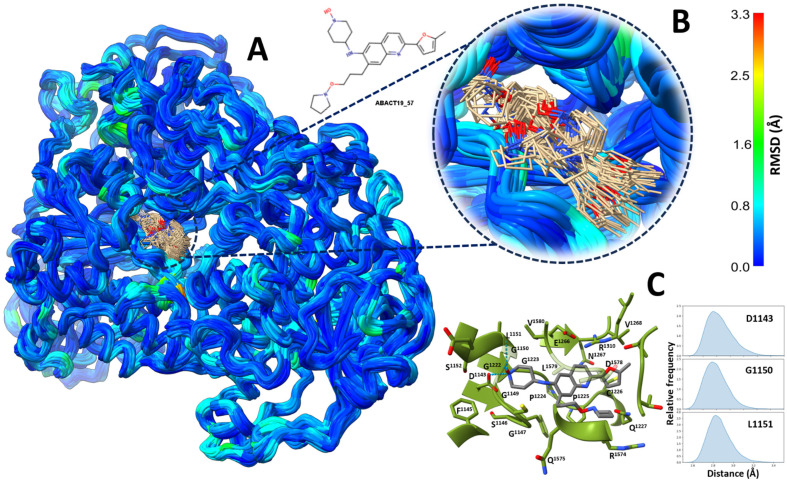
Molecular dynamics simulations of DNMT1 in complex with compound ABACT19_57. **A**) Snapshots from five 100 ns trajectories, taken every 5 ns, were aligned based on a subset of residues exhibiting the lowest backbone RMSD as determined by the MDLovofit program [105]. Regions within each snapshot were color-coded according to the RMSD values of the backbone’s heavy atoms. **B**) Close-up of the catalytic site. For ease of visualization, only conformers taken every 25 ns are shown. **C**) Schematic representation of prevalent protein–ligand interactions. Labeled residues had a cumulative contact frequency of at least 0.95 across all five replicates. The adjacent graphs show the frequency distributions of the distance between donor and acceptor atoms for the three prevalent intermolecular hydrogen bonds. All molecular structure representations were created using UCSF ChimeraX v1.6 [106].

MD simulations in explicit solvent and without constraints were conducted to assess the potential of the designed molecules to function as true binders for DNMT1 [103,104]. Five 100 ns replicas were performed for three enzyme-inhibitor complexes. Figure 19 illustrates the results for the complex with ABACT19_57. Throughout all trajectories, the designed compound maintained a notably constant pose, with only the solvent-exposed oxypyrrolidine group showing significant conformational fluctuations (Figure 19A,B). In the original pose obtained by molecular docking, ABACT19_57 was embedded at the bottom of the catalytic cavity, with the hydroxilamine group hydrogen bonded to the side chain of D1143 and the main chains of G1150 and L1151. These three hydrogen bonds exhibited very high prevalence during the trajectories, as indicated by the distance frequency between the corresponding donor-acceptor atom pairs (Figure 19C). In addition to these polar-to-polar interactions, ABACT19_57 was stabilized by another 21 residues, each exhibiting a cumulative contact frequency greater than 0.95.

ABACT13_40 was another compound with predicted pose-forming intermolecular hydrogen bonds at the bottom of the enzyme’s active site (Figure S2). The side chains of T1528 and R1574 bridged with the oxygen atom of 4-methoxypyridine and the hydroxyl group of the compound, respectively. Furthermore, during the thermalization process, the side chain of N1578 reoriented to form a hydrogen bond with the nitrogen atom of the pyridine. These three hydrogen bonds, similar to those observed with compound ABACT19_57, but involving different residues, showed high prevalence (Figure S2C). Indeed, ABACT13_40, stabilized by highly persistent contacts with 25 residues, exhibited even lower mobility than ABACT19_57. Conversely, the compound ABACT20_12 did not form any stable hydrogen bonds with the enzyme, either in the original pose or during the trajectories, being stabilized by persistent van der Waals contacts with only nine residues (Figure S3). As a result, this compound displayed greater mobility within the enzyme binding site compared to compounds ABACT13_40 and ABACT19_57.

## 4. Conclusions and Perspectives

In this study, two de novo libraries focused on the epigenetic target DNMT1 were designed, with 5575 and 2491 compounds, respectively. The larger library was created with alvaBuilder, a ligand-based program, while the other was constructed with LigBuilder, software based on protein structure. The number of compounds was smaller since the structure-based strategy took more time and computational resources.

The visualization of the chemical spaces showed that de novo compounds kept the physicochemical properties of the already known DNMT1 active inhibitors reported in ChEMBL 31. This could be associated with the secondary constraints used in both strategies for the design, in the scoring function of alvaBuilder and the parameters for the BUILD module in LigBuilder. These results are encouraging, since the visualizations with the two fingerprints used as molecular representations suggest that newly designed compounds populate different areas of the chemical space. Therefore, the new focused libraries are relevant for expanding the chemical space of DNMT1 inhibitors while keeping drug-like properties.

In general, QED and SAscore values for alvaBuilder molecules were alike or better than the reference database (ChEMBL’s DNMT1 inhibitors). Designed molecules from FooDB and UNPD-A exhibit decreased values of both scores, possibly because of the higher molecular complexity of food chemicals and natural products compared to small molecules of synthetic origin. Means from LigBuilder molecules were below the reference database for both QED and SAscore. These relative values could be associated with the penalty of molecular complexity and inherent synthetic feasibility problems related to de novo design [3]. Compounds designed with alvaBuilder were the most diverse based on MACCS Keys, ECFP4 fingerprints, and scaffolds.

Results from the classification evidenced that predicted active molecules with both models are quinolines. Moreover, compounds designed from UNPD-A with both programs had the second-highest proportion of active compounds after those designed from ChEMBL’s DNMT1 inhibitors. This suggests that, even though the QED and SAscore are less favorable for UNPD-A compounds, the activity could be the one searched, making this dataset suitable for the beginning of an optimization project. Finally, there is evidence that the biological activity information from the ChEMBL’s DNMT1 actives is kept, since compounds constructed from them had the highest proportion of actives for both de novo software. Medians of the molecular docking scores of the total of designed molecules had the same trend.

The newly focused libraries developed in this study contain a complete description of the methodology used to construct them and represent a notable addition to the commercial epigenetic-focused libraries currently available. In agreement with open science and its symbiosis with artificial intelligence and other computational applications [107], all fragment libraries generated in this work are freely available and can be used for further virtual and experimental screening for epigenetic drug discovery, emphasizing DNMT1. The ligand- and structure-based de novo design strategies implemented in this work are general and could be used to build screening libraries focused on other epigenetic targets.

Overall, both focused libraries have different profiles and represent valuable starting points for expanding the chemical space of DNMT1 inhibitors. AlvaBuilder and LigBuilder compounds generally preserve molecular properties calculated from known DNMT1 inhibitors. Compounds from the ligand-based strategy are more diverse than those from the LigBuilder ones and exhibit better profiles of QED and SAscore. However, both libraries had similar results with docking scores and had predicted actives with the classification model. Since LigBuilder compounds are restricted to the binding pocket, the diversity could diminish. Meanwhile, alvaBuilder compounds also had higher proportions of actives with the classification models, probably due to the fragment characteristics. This could also translate into more novelty for the LigBuilder molecules. One library or another could be chosen depending on the objective of the discovery project.

Perspectives of this study include the chemical synthesis and testing of the entire compound libraries or selected compounds, and further virtual screening of the de novo-designed libraries to select additional individual compounds for synthesis and testing. Similarity searches in commercial libraries for later acquisition and experimental screening with DNMT1 in enzymatic inhibition and other assays are underway in our research group.

## Figures and Tables

**Table 1:** Training chemical libraries used for alvaBuilder as fragment sources.

Database	Description	Number of Compounds
ChemDiv DNMT-targeted library [42]	Small molecules targeting DNMTs.	33,936
ChemDiv Epigenetics Focused Set [43]	Drug-like compounds targeting families of epigenetic proteins.	25,883
ChemDiv Soluble Diversity Library [44]	Soluble drug-like compounds focused against various biological targets.	15,500
ChEMBL actives [45]	Inhibitors with IC_50_ ≤ 10 μM from ChEMBL 31.	285
FooDB [46]	Food chemicals.	68,658
Life Chemicals Diversity Set [47]	A subset of 5000 drug-like compounds with a wide range of chemical structures.	5120
Life Chemicals Epigenetic Focused Library [48]	Drug-like compounds selected by 2D fingerprint similarity search with known epigenetic modulators.	3578
UNPD-A [49,50]	A diverse subset from the Universal Natural Product Database.	14,994

**Table 3:** Global diversity analysis of the newly designed compound libraries.

Dataset	Code CDP	Size	ECFP4 ^a^	MACCS Keys ^a^	N/M	AUC	F50	SSE10	Molecular Properties
DNMT1	1	743	0.140	0.437	0.634	0.787	0.045	0.927	3.275
alvaBuilder	2	5575	0.148	0.431	0.795	0.734	0.138	0.739	2.396
LigBuilder	3	2491	0.171	0.533	0.501	0.826	0.020	0.939	3.108

M: number of molecules, N: number of scaffolds, AUC: area under the curve, F50: fraction of chemotypes that contain 50% of the dataset, SSE10: Scaled Shannon Entropy at the ten most frequent scaffolds. ^a^ Median of the pairwise fingerprint similarity distribution.

**Table 4:** Distance-to-model performance of classification models on the training set.

Quartile	Number of Active Compounds		SVM + RDK		FFNN + Morgan	
	Active	Inactive	Precision	Recall	Precision	Recall
Q1	44	1	0.978	0.978	0.978	1.000
Q2	40	5	0.974	0.950	0.950	0.950
Q3	20	25	0.667	0.300	0.667	0.600
Q4	9	36	0.667	0.222	0.600	0.333

**Table 5:** Compounds predicted as active by the SVM + RDK model.

Database	Quartile			Proportion
	Q2	Q3	Q4	
alvaBuilder—ChemDiv DNMT	1	6	8	0.024

**Table 6:** Compounds predicted as active by the FFNN + Morgan model.

Database	Quartile			Proportion
	Q2	Q3	Q4	
alvaBuilder				
ChemDiv DNMT	0	0	130	0.186
ChemDiv epigenetics	0	0	110	0.157
ChemDiv soluble	0	0	97	0.139
DNMT1 actives	1	12	141	0.220
FooDB	0	0	97	0.139
Life Chemicals diverse	0	0	132	0.189
Life Chemicals epigenetics	0	0	111	0.159
UNPD-A	0	0	137	0.202
LigBuilder				
ChemDiv epigenetics	0	0	16	0.049
ChemDiv fragments	0	0	3	0.007
ChemDiv soluble	0	0	7	0.024
LigBuilder default	0	0	13	0.042
DNMT1 actives	0	0	37	0.094
FooDB	0	0	7	0.022
Selleckchem	0	0	9	0.027
UNPD-A	0	0	6	0.051

## Data Availability

Data and code related to this work are freely available at https://github.com/DIFACQUIM/, accessed on 25 June 2024.

## Supplementary Materials

The following supporting information can be downloaded at: https://www.mdpi.com/article/10.3390/biom14070775/s1, Table S1. Ranges calculated to establish the scoring function of alvaBuilder; Figure S1. Scaffolds from nucleoside analogs excluded from the alvaBuilder design; Table S2. Remaining compounds after each step of filtration for the alvaBuilder chemical libraries; Table S3. Remaining fragments after each step of filtration for the LigBuilder libraries; Table S4. Optimized hyperparameters for each machine-learning algorithm; Table S5. Descriptive statistics of similarity distribution computed for de novo compounds designed with alvaBuilder. The Tanimoto coefficient was used as the similarity index and MACCS Keys (166-bits) fingerprint as the molecular representation; Table S6. Descriptive statistics of similarity distribution computed for de novo compounds designed with alvaBuilder. The Tanimoto coefficient was used as the similarity index and ECFP4 (1024 bits) fingerprint as the molecular representation; Table S7. Descriptive statistics of similarity distribution computed for de novo compounds designed with LigBuilder. The Tanimoto coefficient was used as the similarity index and MACCS Keys (166-bits) fingerprint as the molecular representation; Table S8. Descriptive statistics of similarity distribution computed for de novo compounds designed with LigBuilder. The Tanimoto coefficient was used as the similarity index and ECFP4 (1024 bits) fingerprint as the molecular representation; Table S9. Ligand efficiency values for alvaBuilder compounds, computed from LeDock scores; Table S10. Ligand efficiency values for alvaBuilder compounds, computed from Vina scores; Table S11. Ligand efficiency values for LigBuilder compounds, computed from LeDock scores; Table S12. Ligand efficiency values for LigBuilder compounds, computed from Vina scores; Table S13. Predictive performance of classification models; Table S14. Distance-to-model performance of classification models on the test set; Figure S2. Molecular dynamics simulations of DNMT1 in complex with compound ABACT13_40; Figure S3. Molecular dynamics simulations of DNMT1 in complex with compound ABACT20_12.

## Abbreviations

3D, three-dimensional; AUC, area under the curve; CDPs, consensus diversity plots; ESOL, estimated solubility; HBAs, hydrogen bond acceptors; HBDs, hydrogen bond donors; DNMT, DNA methyltransferase; ECFP4, Extended Connectivity Fingerprint of radius 2; IC_50_, half-maximal inhibitory concentration; LE, ligand efficiency; logD, distribution coefficient; logP, partition coefficient; MD, molecular dynamics; MOE, Molecular Operating Environment; MW, molecular weight; PC, principal component; nRotB, number of rotatable bonds; PCA, principal component analysis; PDB, Protein Data Bank; QED, quantitative estimate of drug-likeness; RECAP, retrosynthetic combinatorial analysis procedure; RMSD, root mean square deviation; RO3, Rule of Three; SAH, *S*-Adenosyl-*L*-homocysteine; SAscore, synthetic accessibility score; TPSA, topological polar surface area; t-SNE, t-distributed stochastic neighbor embedding; Vina, AutoDock Vina.
