# Supplementary material for: De Novo Design of Inhibitors of DNA Methyltransferase 1: A Critical Comparison of Ligand- and Structure-Based Approaches
Source: Biomolecules. 2024 Jun 28;14(7):775. doi: 10.3390/biom14070775 (PMC11274800; doi:10.3390/biom14070775)
Supplement: Supplementary file 1 [file biomolecules-14-00775-s001.zip › biomolecules-3004567-supplementary.pdf]

Supplementary Materials

De Novo Design of Inhibitors of DNA Methyltransferase 1: A Critical Comparison of Ligand- and Structure-Based Approaches

Diana L. Prado-Romero [1], Fernanda I. Saldívar-González [1], Iván López-Mata [2,3], Pedro A. Laurel-García [1], Adrián Durán-Vargas [4], Enrique García-Hernández [4], Norberto Sánchez-Cruz [3,5] and José L. Medina-Franco [1,*]

Contents	Page
**Table S1.** Ranges calculated to establish the scoring function of alvaBuilder.	S2
**Figure S1.** Scaffolds from nucleoside analogs excluded from the alvaBuilder design.	S2
**Table S2.** Remaining compounds after each step of filtration for the alvaBuilder chemical libraries.	S2
**Table S3.** Remaining fragments after each step of filtration for the LigBuilder libraries.	S3
**Table S4.** Optimized hyperparameters for each machine-learning algorithm.	S3
**Table S5.** Descriptive statistics of similarity distribution computed for de novo compounds designed with alvaBuilder. The Tanimoto coefficient was used as the similarity index and MACCS Keys (166 bits) fingerprint as the molecular representation.	S4
**Table S6.** Descriptive statistics of similarity distribution computed for de novo compounds designed with alvaBuilder. The Tanimoto coefficient was used as the similarity index and ECFP4 (1024 bits) fingerprint as the molecular representation.	S4
**Table S7.** Descriptive statistics of similarity distribution computed for de novo compounds designed with LigBuilder. The Tanimoto coefficient was used as the similarity index and MACCS Keys (166 bits) fingerprint as the molecular representation.	S5
**Table S8.** Descriptive statistics of similarity distribution computed for de novo compounds designed with LigBuilder. The Tanimoto coefficient was used as the similarity index and ECFP4 (1024 bits) fingerprint as the molecular representation.	S5
**Table S9.** Ligand efficiency values for alvaBuilder compounds, computed from LeDock scores.	S6
**Table S10.** Ligand efficiency values for alvaBuilder compounds, computed from Vina scores.	S6
**Table S11.** Ligand efficiency values for LigBuilder compounds, computed from LeDock scores.	S7
**Table S12.** Ligand efficiency values for LigBuilder compounds, computed from Vina scores.	S7
**Table S13.** Predictive performance of classification models.	S8
**Table S14.** Distance-to-model performance of classification models on the test set.	S9
**Figure S2.** Molecular dynamics simulations of DNMT1 in complex with compound ABACT13_40.	S9
**Figure S3.** Molecular dynamics simulations of DNMT1 in complex with compound ABACT20_12.	S10

Table S1. Ranges calculated to establish the scoring function of alvaBuilder.

Descriptor	alvaDesc values (minimum-maximum)	alvaBuilder ranges
**MW**	175.02 – 664.44	351.86 – 539.04
**HBD**	0 – 8	1 – 5
**HBA**	1 – 12	5 – 9
**LogP consensus**	-2.721 – 6.910	1.55 – 4.87
**ESOL**	-7.402 – 0.856	-5.84 – -3.03
**SAscore**	3.096 – 5.979	≤ 5.979
**TPSA**	17.290 – 220.820	60.91 – 152.67

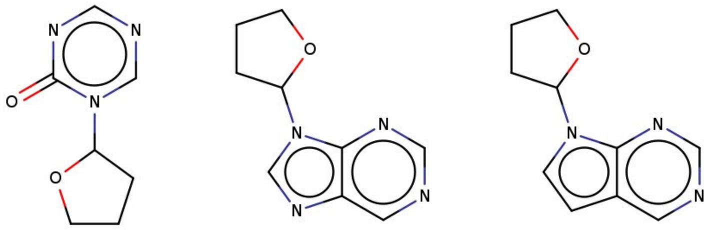

Figure S1. Scaffolds from nucleoside analogs excluded from the alvaBuilder design.

Table S2. Remaining compounds after each step of filtration for the alvaBuilder chemical libraries.

**Data set**	**Original number**	**After curation**	**MW filter**
ChemDiv-DNMT	33 936	33 935	33 006
ChemDiv-Epigenetics	25 883	25 883	25 072
ChemDiv-Soluble	15 500	15 500	8793
DNMT1	316	285	—
FooDB	68 568	52 856*	45 276
Life Chemicals-Diversity	5120	5119	3789
LifeChemicals-Epigenetic	3578	3574	2613
UNPD-A	14 994	14 994	8983

*Less compounds due to exclusion of chirality information.

Table S3. Remaining fragments after each step of filtration for the LigBuilder libraries.

**Data set**	**Original number**	**After curation**	**MW filter**	**ADMET evaluation**	**logD filter**
ChemDiv-Fragments	11 269	11 269	11 195	400	349
ChemDiv-Epigenetics	9196	9196	974	400	311
DNMT1	1645	1569	776	399	246
FooDB	225 206	208 828	13 204	396	322
LifeChemicals-Soluble	1280	1280	1280	400	380
Selleckchem	1015	1012	1012	400	379
UNPD-A	412 110	403 874	16 701	395	318

Table S4. Optimized hyperparameters for each machine-learning algorithm.

Algorithm	Hyperparameter space
**k-NN**	n_beighbors: 1, 3, 5, 7, 9<br>metric: jaccard
**RF**	n_estimators: 1000<br>max_features: 1, 2, 3, 4, 5
**GBT**	n_estimators: 1000<br>max_features: 1, 2, 3, 4, 5<br>max_depth: 4, 5, 6, 7, 8, 9 min_samples_split: 2, 3, 4, 5
**SVM**	C: 0.001, 0.01, 0.1, 1.0, 10.0, 100.0<br>kernel: linear, rbf, sigmoid
**FFNN**	solver: lbfgs<br>max_iter: 10000<br>hidden_layer_sizes: (8,) (16,)<br>activation: logistic, tanh, relu

Table S5. Descriptive statistics of similarity distribution computed for de novo compounds designed with alvaBuilder. The Tanimoto coefficient was used as the similarity index and MACCS Keys (166 bits) fingerprint as the molecular representation.

	ChEMBL actives	ChemDiv DNMT	ChemDiv epigenetics	ChemDiv soluble	DNMT1 actives	FooDB	Life Chemicals diverse	Life Chemicals epigenetics	UNPD-A
**Mean**	0.482	0.462	0.476	0.495	0.495	0.417	0.443	0.466	0.399
**Std**	0.179	0.095	0.096	0.088	0.112	0.106	0.092	0.098	0.110
**Min**	0.037	0.113	0.107	0.111	0.076	0.026	0.096	0.059	0.039
**Q1**	0.357	0.397	0.410	0.436	0.419	0.346	0.380	0.400	0.324
**Median (Q2)**	0.455	0.461	0.474	0.494	0.490	0.415	0.440	0.465	0.397
**Q3**	0.570	0.525	0.539	0.554	0.568	0.484	0.500	0.531	0.470
**Max**	1	1	1	1	0.986	1	1	1	1

Table S6. Descriptive statistics of similarity distribution computed for de novo compounds designed with alvaBuilder. The Tanimoto coefficient was used as the similarity index and ECFP4 (1024 bits) fingerprint as the molecular representation.

	ChEMBL actives	ChemDiv DNMT	ChemDiv epigenetics	ChemDiv soluble	DNMT1 actives	FooDB	Life Chemicals diverse	Life Chemicals epigenetics	UNPD-A
**Mean**	0.171	0.145	0.146	0.139	0.161	0.124	0.134	0.144	0.121
**Std**	0.156	0.046	0.046	0.043	0.068	0.052	0.044	0.046	0.059
**Min**	0	0.018	0.027	0.019	0.010	0	0.010	0.028	0
**Q1**	0.092	0.116	0.118	0.113	0.118	0.096	0.107	0.115	0.091
**Median (Q2)**	0.116	0.139	0.140	0.135	0.146	0.118	0.13	0.138	0.115
**Q3**	0.152	0.167	0.167	0.158	0.184	0.143	0.155	0.165	0.14
**Max**	0.984	0.905	0.912	0.923	0.917	0.967	0.864	0.879	1

Table S7. Descriptive statistics of similarity distribution computed for de novo compounds designed with LigBuilder. The Tanimoto coefficient was used as the similarity index and MACCS Keys (166 bits) fingerprint as the molecular representation.

	ChEMBL actives	LigBuilder default	ChemDiv epigenetics	ChemDiv fragments	DNMT1 actives	FooDB	Life Chemicals soluble	Selleckchem	UNPD-A
**Mean**	0.482	0.533	0.576	0.587	0.548	0.584	0.589	0.512	0.511
**Std**	0.179	0.099	0.094	0.091	0.122	0.1	0.108	0.118	0.107
**Min**	0.037	0.108	0.192	0.197	0.111	0.176	0.225	0.188	0.152
**Q1**	0.357	0.469	0.518	0.529	0.476	0.52	0.519	0.426	0.439
**Median (Q2)**	0.455	0.529	0.577	0.582	0.553	0.581	0.578	0.507	0.506
**Q3**	0.57	0.59	0.633	0.639	0.622	0.645	0.644	0.587	0.574
**Max**	1	1	1	1	1	1	1	1	1

Table S8. Descriptive statistics of similarity distribution computed for de novo compounds designed with LigBuilder. The Tanimoto coefficient was used as the similarity index and ECFP4 (1024 bits) fingerprint as the molecular representation.

	ChEMBL actives	LigBuilder default	ChemDiv epigenetics	ChemDiv fragments	DNMT1 actives	FooDB	Life Chemicals soluble	Selleckchem	UNPD-A
**Mean**	0.171	0.157	0.153	0.176	0.161	0.168	0.173	0.279	0.168
**Std**	0.156	0.075	0.071	0.087	0.092	0.085	0.104	0.114	0.08
**Min**	0	0.040	0.043	0.049	0.028	0.048	0.049	0.071	0.047
**Q1**	0.092	0.123	0.123	0.133	0.115	0.127	0.127	0.208	0.126
**Median (Q2)**	0.116	0.144	0.142	0.155	0.140	0.148	0.147	0.260	0.151
**Q3**	0.152	0.169	0.164	0.187	0.171	0.174	0.171	0.312	0.181
**Max**	0.984	0.982	0.938	1	1	0.929	1	0.875	0.941

Table S9. Ligand efficiency values for alvaBuilder compounds, computed from LeDock scores.

	ChEMBL actives	ChemDiv DNMT	ChemDiv epigenetics	ChemDiv soluble	DNMT1 actives	FooDB	Life Chemicals diverse	Life Chemicals epigenetics	UNPD-A
**Total of compounds**	285	699	700	700	700	699	700	700	677
**Mean**	-0.262	-0.272	-0.279	-0.259	-0.267	-0.243	-0.272	-0.265	-0.256
**Std**	0.048	0.039	0.039	0.038	0.035	0.038	0.041	0.038	0.040
**Min**	-0.448	-0.442	-0.429	-0.398	-0.401	-0.413	-0.432	-0.401	-0.416
**Q1**	-0.283	-0.298	-0.295	-0.284	-0.287	-0.266	-0.295	-0.289	-0.280
**Median (Q2)**	-0.251	-0.270	-0.267	-0.257	-0.267	-0.242	-0.268	-0.264	-0.254
**Q3**	-0.231	-0.245	-0.244	-0.233	-0.243	-0.219	-0.244	-0.238	-0.226
**Max**	-0.117	-0.177	-0.164	-0.162	-0.177	-0.106	-0.169	-0.158	-0.139

Table S10. Ligand efficiency values for alvaBuilder compounds, computed from Vina scores.

	ChEMBL actives	ChemDiv DNMT	ChemDiv epigenetics	ChemDiv soluble	DNMT1 actives	FooDB	Life Chemicals diverse	Life Chemicals epigenetics	UNPD-A
**Total of compounds**	285	699	700	700	700	699	700	700	677
**Mean**	-0.296	-0.298	-0.295	-0.286	-0.287	-0.271	-0.295	-0.296	-0.270
**Std**	0.061	0.039	0.041	0.041	0.037	0.045	0.039	0.038	0.044
**Min**	-0.557	-0.411	-0.417	-0.402	-0.404	-0.423	-0.413	-0.407	-0.399
**Q1**	-0.322	-0.325	-0.325	-0.314	-0.311	-0.304	-0.320	-0.322	-0.298
**Median (Q2)**	-0.284	-0.298	-0.294	-0.284	-0.286	-0.268	-0.294	-0.295	-0.267
**Q3**	-0.255	-0.268	-0.266	-0.255	-0.260	-0.260	-0.268	-0.270	-0.240
**Max**	-0.170	-0.203	-0.186	-0.189	-0.185	-0.185	-0.174	-0.194	-0.159

Table S11. Ligand efficiency values for LigBuilder compounds, computed from LeDock scores.

	ChEMBL actives	LigBuilder default	ChemDiv epigenetics	ChemDiv fragments	DNMT1 actives	FooDB	Life Chemicals soluble	Selleckchem	UNPD-A
**Total of compounds**	285	309	329	403	395	315	292	331	117
**Mean**	-0.262	-0.304	-0.297	-0.289	-0.294	-0.301	-0.288	-0.276	-0.283
**Std**	0.048	0.040	0.037	0.046	0.039	0.040	0.038	0.029	0.041
**Min**	-0.448	-0.433	-0.422	-0.512	-0.472	-0.462	-0.440	-0.370	-0.426
**Q1**	-0.283	-0.328	-0.319	-0.317	-0.318	-0.326	-0.308	-0.296	-0.301
**Median (Q2)**	-0.251	-0.300	-0.292	-0.288	-0.289	-0.299	-0.284	-0.276	-0.278
**Q3**	-0.231	-0.279	-0.270	-0.252	-0.266	-0.273	-0.263	-0.254	-0.255
**Max**	-0.117	-0.215	-0.210	-0.177	-0.186	-0.194	-0.186	-0.199	-0.204

Table S12. Ligand efficiency values for LigBuilder compounds, computed from Vina scores.

	ChEMBL actives	LigBuilder default	ChemDiv epigenetics	ChemDiv fragments	DNMT1 actives	FooDB	Life Chemicals soluble	Selleckchem	UNPD-A
**Total of compounds**	285	309	329	403	395	315	292	331	117
**Mean**	-0.296	-0.292	-0.273	-0.294	-0.280	-0.288	-0.269	-0.295	-0.298
**Std**	0.061	0.042	0.037	0.043	0.040	0.041	0.043	0.035	0.048
**Min**	-0.557	-0.417	-0.408	-0.446	-0.421	-0.389	-0.407	-0.416	-0.444
**Q1**	-0.322	-0.322	-0.298	-0.324	-0.309	-0.319	-0.295	-0.316	-0.326
**Median (Q2)**	-0.284	-0.287	-0.272	-0.293	-0.274	-0.287	-0.261	-0.293	-0.298
**Q3**	-0.255	-0.258	-0.247	-0.263	-0.251	-0.255	-0.240	-0.269	-0.264
**Max**	-0.170	-0.191	-0.189	-0.194	-0.200	-0.194	-0.176	-0.225	-0.191

Table S13. Predictive performance of classification models.

Model	Best hyperparameters	BA Train	BA Test
**SVM + RDK**	{'C': 1.0, 'kernel': 'rbf', 'probability': True}	0.849	0.775
**SVM + Morgan**	{'C': 10.0, 'kernel': 'rbf', 'probability': True}	0.849	0.675
**FFNN + Morgan**	{'activation': 'tanh', 'hidden_layer_sizes': (16,), 'max_iter': 10000, 'solver': 'lbfgs'}	0.847	0.793
**GBT + RDK**	{'max_depth': 7, 'max_features': 3, 'min_samples_split': 4, 'n_estimators': 1000}	0.845	0.723
**RF + RDK**	{'max_features': 3, 'n_estimators': 1000}	0.845	0.723
**k-NN + RDK**	{'metric': 'jaccard', 'n_neighbors': 3}	0.833	0.711
**k-NN + Morgan**	{'metric': 'jaccard', 'n_neighbors': 3}	0.833	0.646
**GBT + Morgan**	{'max_depth': 5, 'max_features': 1, 'min_samples_split': 2, 'n_estimators': 1000}	0.82	0.687
**GBT + MACCS**	{'max_depth': 6, 'max_features': 5, 'min_samples_split': 2, 'n_estimators': 1000}	0.807	0.705
**RF + Morgan**	{'max_features': 2, 'n_estimators': 1000}	0.807	0.705
**FFNN + RDK**	{'activation': 'tanh', 'hidden_layer_sizes': (8,), 'max_iter': 10000, 'solver': 'lbfgs'}	0.804	0.734
**k-NN + MACCS**	{'metric': 'jaccard', 'n_neighbors': 5}	0.801	0.723
**FFNN + MACCS**	{'activation': 'tanh', 'hidden_layer_sizes': (16,), 'max_iter': 10000, 'solver': 'lbfgs'}	0.786	0.675
**SVM + MACCS**	{'C': 10.0, 'kernel': 'rbf', 'probability': True}	0.782	0.646
**RF + MACCS**	{'max_features': 5, 'n_estimators': 1000}	0.776	0.675

Table S14. Distance-to-model performance of classification models on the test set.

Quartile	Number of active compounds		SVM + RDK		FFNN + Morgan	
	Active	Inactive	Precision	Recall	Precision	Recall
Q1	10	0	1.0	1.0	1.0	1.0
Q2	9	1	0.9	0.9	0.9	1.0
Q3	5	10	0.5	0.6	0.4	0.4
Q4	4	6	1.0	0.25	1.0	0.25

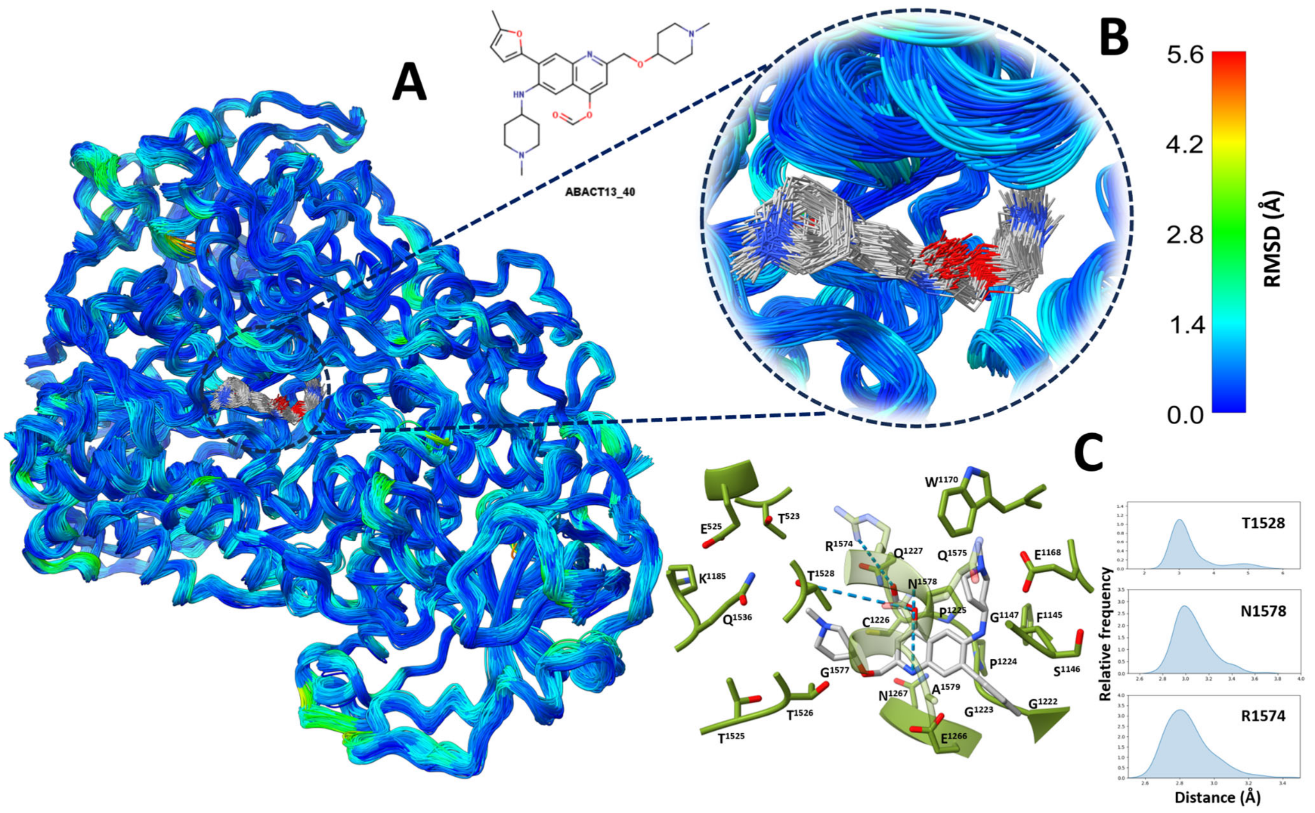

Figure S2. Molecular dynamics simulations of DNMT1 in complex with compound ABACT13_40. **A.** Snapshots from five 100-ns trajectories, taken every 5 ns, were aligned based on a subset of residues exhibiting the lowest backbone RMSD as determined by the MDLovofit program [105]. Regions within each snapshot were color-coded according to the RMSD values of the backbone's heavy atoms. **B.** Close-up of the catalytic site. For ease of visualization, only conformers taken every 25 ns are shown. **C.** Schematic representation of prevalent protein-ligand interactions. Labeled residues had a cumulative contact frequency of at least 0.95 across all five replicates. The adjacent graphs show the frequency distributions of the distance between donor and acceptor atoms for the three prevalent intermolecular hydrogen bonds. All molecular structure representations were created using UCSF ChimeraX v1.6.

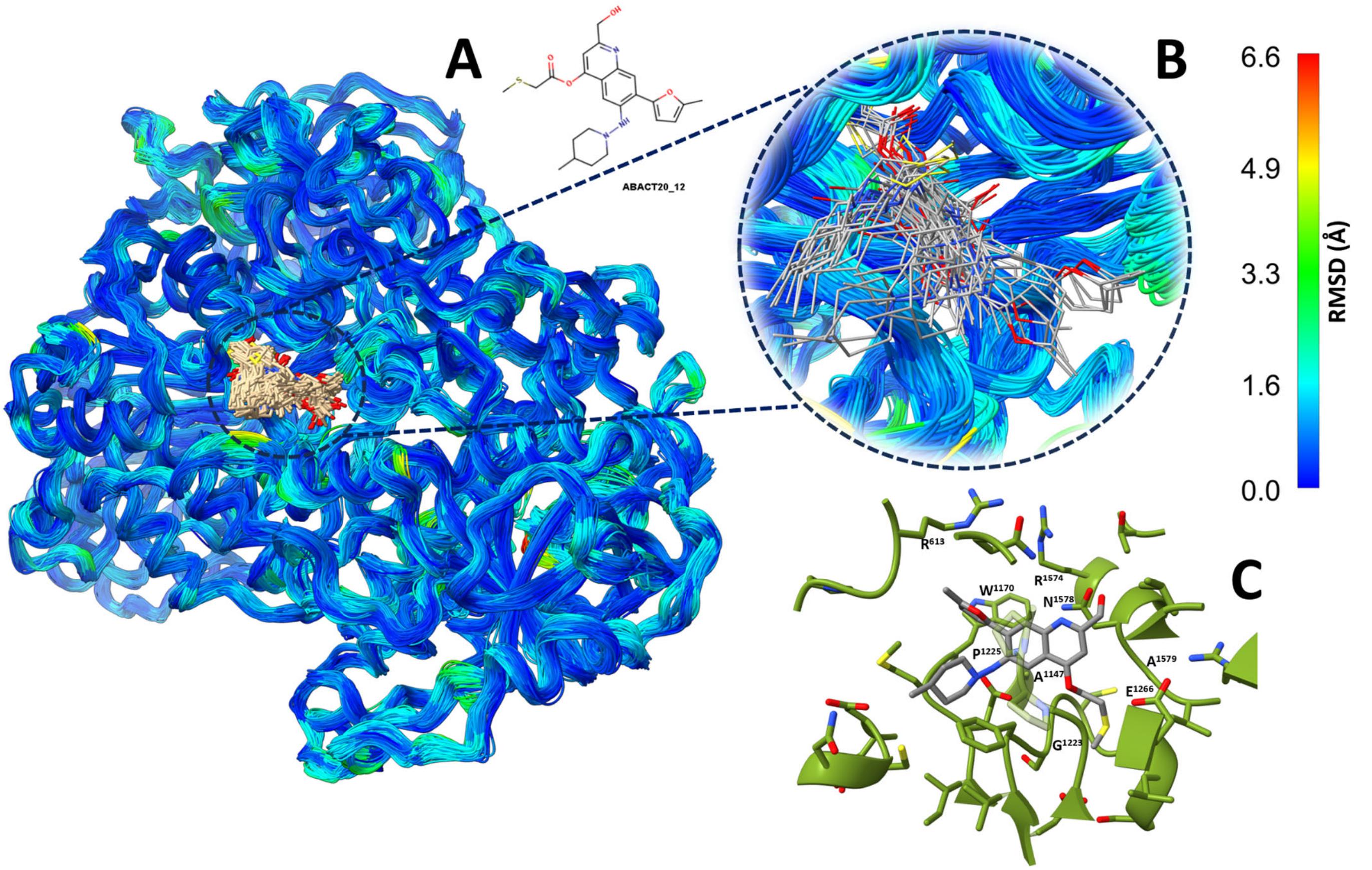

Figure S3. Molecular dynamics simulations of DNMT1 in complex with compound ABAC20_12. **A.** Snapshots from five 100-ns trajectories, taken every 5 ns, were aligned based on a subset of residues exhibiting the lowest backbone RMSD as determined by the MDLovofit program [105]. Regions within each snapshot were color-coded according to the RMSD values of the backbone's heavy atoms. **B.** Close-up of the catalytic site. For ease of visualization, only conformers taken every 25 ns are shown. **C.** Schematic representation of prevalent protein-ligand interactions. Labeled residues had a cumulative contact frequency of at least 0.95 across all five replicates. All molecular structure representations were created using UCSF ChimeraX v1.6.
